# Thermotherapy for Chronic Wounds: A Wound-Interface Translation Framework for Materials, Devices, Clinical Evidence, and Validation Requirements

**DOI:** 10.3390/bioengineering13101126

**Published:** 2026-09-27

**Authors:** Sameer Ahmad Hasan, Bassam Al-Naami, Muhammad Al-Ayyad, Abdel-Razzak Al-Hinnawi, Olga Korostynska

**Affiliations:** 1 Department of Biomedical Engineering, School of Applied Medical Sciences, German Jordanian University, Amman 11180, Jordan; sameer.hasan@gju.edu.jo; 2 Department of Biomedical Engineering, Faculty of Engineering, The Hashemite University, Zarqa 13133, Jordan; b.naami@hu.edu.jo; 3 Department of Medical Engineering, Faculty of Engineering, Al-Ahliyya Amman University, Amman 19328, Jordan; mayyad@ammanu.edu.jo; 4 Department of Radiographic Technology, Faculty of Allied Medical Sciences, Isra University, Amman 11622, Jordan; abedalrazak.henawai@iu.edu.jo; 5 ADEPT Research Group, Department of Mechanical, Electrical and Chemical Engineering, Faculty of Technology, Art and Design, Oslo Metropolitan University, 0166 Oslo, Norway

**Keywords:** bioresorbable heaters, chronic wounds, closed-loop control, flexible electronics, photothermal materials, smart dressings, thermal dose, thermotherapy

## Abstract

Interest in local warming for chronic wound care has re-emerged as flexible heaters, embedded sensors, and smart dressings make controlled heat delivery increasingly feasible. This narrative translational review treats thermotherapy as a dose-dependent wound-interface intervention rather than a single warming technology. It distinguishes normothermic radiant warming, water-filtered infrared A, resistive Joule heating, photothermal systems, and temperature-monitoring technologies by mechanism, thermal context, therapeutic purpose, and evidence level. The review integrates wound pathophysiology, biological responses to temperature, thermal-dose concepts, emerging heater platforms, sensing and closed-loop control, clinical evidence, safety, and regulatory translation. Direct human treatment evidence remains concentrated in small historical radiant-warming and water-filtered infrared A studies, with limited additional local-heat evidence, and cannot be transferred directly to contemporary flexible, wireless, or photothermal platforms. For lower-temperature warming, heater setpoint alone is an inadequate dose descriptor, and the biological meaning of cumulative equivalent minutes at 43 °C (CEM43) has not been validated; measured time–temperature profiles, peak temperature, spatial variation, sensor location, and uncertainty are more informative. Clinical translation therefore depends on reproducible wound-interface dosing under realistic moisture, pressure, perfusion, dressing, motion, and workflow conditions, together with wound-specific dose models, transparent reporting, realistic interface validation, and architecture-specific regulatory evidence.

## 1. Introduction

Chronic wounds remain a substantial clinical and economic problem. Impaired perfusion, infection, and dysregulated matrix remodeling can slow repair or stop it altogether. Their prevalence is increasing with population aging and with the growing burden of diabetes, vascular disease, and immobility [1]. For patients and health systems, the consequences are considerable. Chronic wounds often require repeated clinic visits, hospital admissions, prolonged treatment, and substantial expenditure [2]. Current care is built around debridement, infection control, moisture balance, offloading, and correction of vascular disease where possible [3].

Nevertheless, many ulcers fail to heal even when these measures are optimized. This persistent gap points to an unmet need for adjunctive strategies that address microvascular and cellular dysfunction [4]. The biological rationale for controlled local warming is grounded in several processes involved in repair. Higher cutaneous temperature can increase microvascular blood flow [5] and tissue oxygen tension [6], while thermal stress can increase heat shock protein 70 (HSP70) and inflammatory markers in burn-blister fluid [7]. These effects make thermotherapy a plausible adjunct for selected chronic wounds. The clinical evidence, however, is still uneven. In high-risk settings, particularly ischemic, neuropathic, or infected tissue, safe and effective thermal dosing remains poorly defined. Recent advances in flexible materials and embedded electronics have changed what is technically possible for local heat delivery. Flexible liquid-metal heaters [8], graphene-based conductors [9], and hydrogel-integrated warming systems may provide more conformal and spatially uniform heat transfer than conventional rigid devices [10]. Temperature sensors combined with feedback electronics can adjust power output when contact pressure or ambient conditions change [11]. These features are relevant to chronic wounds because dressings must fit irregular surfaces, tolerate moisture and exudate, and sometimes function under intermittent compression. Early clinical and preclinical studies suggest that local warming may improve perfusion and some healing-related outcomes in chronic wounds, particularly venous leg ulcers, pressure injuries, and related nonhealing wounds [12,13,14]. These observations should be interpreted cautiously. Most studies are small, use diverse patient groups and treatment schedules, and report limited information on achieved interface temperature, temperature stability, spatial heat distribution, or cumulative thermal dose. This makes comparison across studies difficult and prevents confident definition of indications, contraindications, or safe dose ranges. Translation also requires regulatory and standards pathways that can accommodate multifunctional, digitally connected dressings. Existing regulations for powered heating pads [15] and standards for medical heating devices, including blankets, pads, and mattresses [16], mainly address short-term warming of intact skin. They do not fully capture the behavior of flexible, sensor-guided systems used for prolonged periods in moist and potentially contaminated wound environments. Developers therefore need evidence of long-term stability, moisture resistance, and reproducible thermal performance under realistic mechanical loading and microclimatic conditions [17]. Many prototypes are still tested on simplified bench models. Such models do not reproduce the dynamic wound interface, leaving an important gap between engineering performance claims and expected clinical behavior.

### 1.1. Search Strategy, Eligibility, and Evidence Appraisal

This narrative translational review used iterative, topic-specific literature searches from December 2025 through August 2026 across PubMed, Embase, Web of Science Core Collection, and IEEE Xplore. Searches were updated as the review themes were refined, and the evidence base was finalized with a literature cutoff of 31 August 2026. Supplementary Table S1 provides consolidated database-specific search expressions that operationalize the documented search concepts and support reproducible rerunning of the search framework.

The search concepts covered chronic wounds, diabetic foot ulcers, venous leg ulcers, pressure injuries, local warming or thermotherapy, noncontact normothermic wound therapy, thermal dose or CEM43, flexible heaters, smart dressings, photothermal systems, sensing and feedback control, and medical-device regulation. Searches were supplemented by backward citation tracking and by standards, guidance documents, and the device-oriented translational literature when directly relevant to safety, validation, or regulatory interpretation. Reproducibility is supported through the consolidated database-specific expressions in Supplementary Table S1, while the focused clinical selection audit is reported at candidate-report level in Table 1 and Supplementary Figure S1.

For the focused clinical-evidence synthesis, reports were eligible for the core direct-treatment category when they involved human participants with an established chronic wound, evaluated a defined local thermal intervention intended as part of treatment, and reported wound-healing or directly wound-related clinical or physiological outcomes. Studies of intrinsically multimodal thermal technologies, such as water-filtered infrared A (wIRA), were eligible when the thermal intervention was a defined component of treatment, with outcomes interpreted according to the combined modality when thermal and nonthermal effects were inseparable. Studies combining local heating with an independent active co-intervention were classified as supportive/contextual evidence when the contribution of heating could not be evaluated separately. Prevention-only perioperative warming, monitoring-only studies, procedural warming, acute/postoperative wounds outside the chronic-wound treatment scope, and preclinical or bench studies were classified outside the core direct-treatment category. Clinically relevant reports were retained as supportive or contextual evidence where appropriate.

The screening workflow used first-author primary literature searching, initial screening, and eligibility assessment, followed by verification of the final clinical-study selection and classification decisions by two senior coauthors. Points requiring clarification were resolved through discussion. This workflow provided a documented primary assessment with senior coauthor verification appropriate to the narrative translational review design.

Twenty-five unique human reports entered the focused report-level clinical verification. Sixteen met the criteria for the core direct-treatment synthesis, seven were retained as supportive/contextual clinical evidence, one prevention-only report was retained as contextual evidence, and one report was classified outside thermotherapy evidence because it did not evaluate a therapeutic thermal intervention. These four mutually exclusive dispositions account for all 25 candidate reports. The counts represent the focused report-level clinical evidence set. Table 1 gives the disposition and study-specific rationale for each report, and Supplementary Figure S1 summarizes the focused selection flow.

The retained clinical reports were appraised at the study level for assignment method, unit of analysis, baseline comparability, attrition, co-interventions, temperature or dose measurement, adverse-event reporting, and outcome completeness. The evidence tables report only information directly supported by the accessible publication record, with unavailable items identified explicitly. Given the substantial heterogeneity in wound type, intervention modality, treatment schedule, outcome definition, and thermal-dose reporting, the synthesis was qualitative rather than meta-analytic. Generative AI assistance during manuscript preparation was used for language refinement, structural organization, reference-formatting assistance, internal consistency checks, and development of conceptual figure drafts under author guidance; all AI-assisted outputs were reviewed and edited by the authors before incorporation into the manuscript.

### 1.2. Review Scope and Translational Framework

The previous literature has addressed different components of thermotherapy translation largely in separate streams. Reviews of local warming have focused mainly on clinical efficacy and the limited chronic-wound treatment evidence [14,41]. The hyperthermia literature provides established temperature–time concepts such as CEM43, but these frameworks were developed principally for oncologic hyperthermia and their biological meaning for lower-temperature chronic-wound warming remains unvalidated [42,43]. In parallel, the recent smart-bandage and multifunctional-dressing literature has emphasized flexible materials, sensing, feedback control, wireless operation, and multimodal therapy [44,45,46,47], but these engineering capabilities do not by themselves establish a clinically justified wound-interface thermal dose. The present review brings these strands together through five linked questions: whether a thermal exposure can be defined, delivered, measured, controlled, and clinically justified across wound biology, material platform, interface metrology, clinical evidence quality, and regulatory verification. It also separates thermal modalities that differ in energy delivery and biological co-effects, distinguishes direct human treatment evidence from preclinical and monitoring analogies, and proposes wound-interface validation and reporting requirements. The purpose is translational synthesis rather than a pooled estimate of treatment effectiveness.

### 1.3. Chronic Wound Types and Pathophysiologic Barriers to Healing

Chronic wounds are clinically heterogeneous. The principal groups relevant to this review are venous leg ulcers, pressure injuries, diabetes-related foot ulcers, and arterial or mixed-etiology ulcers [1,4,14]. Their failure to heal arises from different initiating causes but commonly involves impaired perfusion or oxygen delivery, persistent inflammation, repeated mechanical loading or venous hypertension, cellular dysfunction, protease-dominant matrix degradation, and susceptibility to infection [4,48,49]. Diabetes-related foot ulcers additionally combine neuropathy, repetitive pressure, and variable ischemia; pressure injuries are driven by sustained or repetitive load and tissue deformation; venous ulcers occur in a milieu of venous hypertension and edema; and arterial or mixed ulcers may have limited capacity to increase perfusion when local metabolic demand rises. These distinctions are central to thermotherapy because the same external heat input can produce different tissue temperatures, heat-dissipation rates, and safety margins across wound types.

Refractory wounds should therefore not be treated as a uniform “cold wound” phenotype. Hypoxia can coexist with inflammation, edema, infection, neuropathy, or impaired vascular reserve, while surface temperature is also influenced by perfusion, ambient exposure, dressings, and inflammatory activity. Any thermotherapeutic rationale must remain wound-specific and adjunctive to etiologic care such as compression, offloading, vascular assessment, debridement, infection management, and moisture balance [4,50].

## 2. Biological Rationale and Biophysical Constraints of Thermotherapy in Chronic Wounds

### 2.1. Terminology, Temperature Ranges, and Temporal Context

Terminology in this field is inconsistent, so this review uses operational definitions rather than implying universally validated chronic-wound thresholds. “Normothermic wound warming” refers to restoring or maintaining the wound or periwound region near physiological tissue temperature; for synthesis in this review, approximately 36–38 °C is used as the normothermic/supportive-warming context, consistent with historical NNWT systems that commonly used a dressing target near 38 °C [24,26]. “Mild thermotherapy” is used here as the broader umbrella term for controlled local warming approximately 36–40 °C when the intended purpose is physiological support. For consistency within this review, “mild hyperthermia” refers operationally to the higher local-warming context of approximately 39–42 °C; broader hyperthermia literature may use wider ranges, including approximately 39–45 °C [51]. These temperature ranges overlap by design and are descriptive contexts rather than validated chronic-wound therapeutic windows. “Photothermal therapy” is defined by its mechanism, namely conversion of optical energy into heat, rather than by a fixed temperature range; the achieved temperature depends on the material, irradiance, exposure duration, and treatment purpose, and photothermal systems may also include nonthermal mechanisms such as photodynamic effects [52,53].

These temperature contexts are not biologically equivalent. Near-normothermic warming (~36–38 °C) primarily supports restoration of physiological tissue conditions and the rationale for perfusion and oxygen delivery, whereas higher local warming (~39–42 °C) can produce more pronounced vasodilation and engage temperature- and time-dependent heat-shock and immunomodulatory responses. With further temperature elevation or prolonged exposure, thermal stress and tissue-injury mechanisms become increasingly important. These ranges should therefore be interpreted as overlapping exposure contexts rather than fixed thresholds for angiogenesis, re-epithelialization, or matrix remodeling.

Temperature also changes during healing. In uncomplicated acute or surgical wounds, inflammatory activity is generally associated with an early temperature elevation, followed by progressive normalization as inflammation resolves and repair proceeds; a secondary rise may accompany infection [54]. Chronic wounds may not follow this trajectory because persistent inflammation, infection, ischemia, edema, neuropathy, and impaired perfusion can produce sustained, reduced, or spatially heterogeneous temperatures. Accordingly, healing phase should not be inferred from an absolute temperature alone; longitudinal wound and periwound measurements are more informative [54,55].

### 2.2. Vascular and Metabolic Responses to Warming

Perfusion is essential for oxygen and nutrient delivery, leukocyte trafficking, and matrix deposition during wound repair. Local warming causes cutaneous vasodilation through neurogenic and nitric oxide-mediated mechanisms. Increased blood flow may raise tissue oxygen tension, which is particularly relevant in chronic wounds where limited oxygen availability impairs collagen synthesis and fibroblast activity. Studies of localized warming in chronic wounds have reported improved periwound perfusion and oxygenation, with greater granulation in some settings [56]. Most of these studies, however, assess only superficial periwound tissue, and whether warming meaningfully affects deeper hypoxic wound regions remains uncertain. Vascular effects are closely linked to temperature-dependent changes in cellular metabolism and oxygen use. Mild warming can increase mitochondrial activity and accelerate biochemical processes involved in cell proliferation and matrix production, while cutaneous warming may further support tissue metabolism through nitric oxide release and endothelial activity [57]. These responses may be relevant in diabetic wounds, where fibroblast and immune-cell dysfunction can impair repair [58]. Their clinical significance remains uncertain because the available wound studies are limited and heterogeneous [41,56]. A 2025 protocol proposed a systematic review of vasomotor and sudomotor responses to increased core temperature in type 2 diabetes; because it is a protocol, it does not itself provide outcome evidence [59].

### 2.3. Cellular Stress, Immune Modulation, and Extracellular Matrix Dynamics

Heat also engages cellular stress-response pathways and can influence innate immune activity. Thermal stimulation can induce heat shock proteins, including HSP70, which stabilize proteins, limit aggregation, and support cell survival under stress. Increased HSP70 expression has been observed in thermally stressed skin, including burn-blister fluid and heat-shocked endothelial cells [7,60]. Post-translational modifications also participate in cellular heat-stress responses, although this evidence is not specific to chronic wounds [61]. These chaperone responses may help protect cells exposed to oxidative stress, inflammation, or mechanical strain in chronic wounds. Even so, the timing of heat-shock induction in chronic wound tissue is not well established, and the temperature–time combinations that provide cytoprotection without protein denaturation remain unclear [56]. Immune dysregulation is a defining feature of chronic wounds. Persistent pro-inflammatory signaling can keep macrophages in inflammatory phenotypes and delay the transition to reparative programs needed for resolution and tissue remodeling [62]. In experimental models, controlled mild warming has shifted macrophage polarization toward reparative, M2-like activity and improved wound repair. Some thermally delivered therapies have increased M2 markers and accelerated closure in diabetic wounds [63]. Thermal exposure can also alter innate immune signaling in a temperature-dependent manner, including changes in Toll-like receptor 4 (TLR4) expression and signaling under fever-range hyperthermia [64]. These changes could help move wounds away from persistent inflammation and toward healing. Still, immune responses to warming are likely to differ by tissue type, infection status, and comorbid disease. Findings from isolated cells or acute models may not reflect the inflammatory complexity of chronic wounds. Matrix remodeling is required for wound closure and depends on coordinated extracellular-matrix turnover by proteases and their endogenous inhibitors. Chronic wounds often have protease-dominant microenvironments, in which elevated matrix metalloproteinase (MMP) activity sustains matrix breakdown and delays healing [48]. Temperature can influence matrix-degrading pathways. Experimental skin studies show that repeated heat exposure can increase several MMPs and promote collagen and extracellular-matrix damage [65]. Because excessive proteolysis is already common in chronic wounds, poorly controlled thermotherapy could worsen destructive matrix degradation. The effect will likely depend on the baseline balance between proteases and their inhibitors. Future studies should therefore report a simple biomarker context, such as the ratio of MMPs to tissue inhibitors of metalloproteinases (TIMPs), together with clinical outcomes [48].

### 2.4. Heat Transfer at the Wound Interface and Implications for Local Temperature Control

The therapeutic effect of thermotherapy depends on heat transfer from the device, across the wound surface, and into deeper tissue. Tissue water content, local blood flow, and the thermal properties of dressings together determine how fast and how deeply tissue warms [66]. Moist granulation tissue under occlusive or hydrogel dressings conducts heat more efficiently than dry or scaly skin. Exudative areas may therefore reach higher temperatures than adjacent regions. Hydrogel dressings used at pressure-prone interfaces also alter contact and local moisture conditions [67]. Many bioheat models used for cutaneous heating originate from intact or homogeneous perfused-tissue assumptions [68]. Chronic wounds rarely satisfy these assumptions. Hydration is uneven, perfusion varies, deeper structures may be exposed, and irregular contours can produce complex temperature gradients over space and time. Clinical thermography of pressure ulcers further demonstrates spatial heterogeneity in wound and periwound temperatures [69]. Current models therefore provide only approximate guidance for chronic-wound thermotherapy and require wound-specific adaptation and validation. These mechanistic and interface uncertainties explain the need for wound-specific dose definitions and measurement requirements, as discussed in Section 3.

## 3. Thermal Dose Concepts and Biophysical Models for Chronic Wounds

### 3.1. Thermal Dose Frameworks: Foundations and Limitations

Thermal dose describes the combined effect of temperature and exposure time on tissue response. CEM43 was developed in oncology hyperthermia to normalize variable time–temperature histories to equivalent minutes at 43 °C [42,43]. In the conventional discrete formulation:

(1) $${CEM}_{43}=\sum_{i}\Delta t_{i}\;R^{\left(43-T_{i}\right)}$$

where Δ*t_i_* is the duration of interval *i* in minutes, *T_i_* is the measured temperature (°C) at the specified measurement site during that interval, and *R* is the empirical time–temperature factor, conventionally 0.5 for *T_i_* > 43 °C and 0.25 for *T_i_* < 43 °C [42,43,51]. At *T_i_* = 43 °C, the exponent is zero, so each minute contributes one equivalent minute. The discrete calculation therefore assumes that *T_i_* adequately represents the measured temperature over each interval and that interval contributions are additive after conversion to the 43 °C reference. It requires a measured temperature–time history from a stated measurement location; a nominal heater setpoint should not be substituted for *T_i_*. The metric is rooted in heat-induced cytotoxicity and does not explicitly represent perfusion-mediated oxygenation or other lower-temperature physiological effects [51]. This limitation matters for chronic-wound interventions that may target near-normothermic temperatures rather than cytotoxic hyperthermia; historical NNWT studies, for example, used a dressing target of 38 °C [24,26]. A CEM43 value can be calculated mathematically at such temperatures, but its biological interpretation as a wound-healing dose has not been validated. Accordingly, CEM43 should not be used as the sole dose descriptor for normothermic wound warming.

Any dose estimate must specify measurement location because heater setpoint, heater-surface temperature, dressing-interface temperature, wound-bed surface temperature, periwound skin temperature, and deeper tissue temperature describe different thermal quantities. Where CEM43 is reported, the underlying temperature–time record and sensor location should accompany the value. For lower-temperature wound warming, the primary dose description should be based on the measured thermal exposure itself: complete time–temperature profile, peak temperature, exposure duration, spatial range or gradient, measurement location, sensor accuracy or uncertainty, and relevant dressing and perfusion conditions. CEM43 can be reported secondarily as a hyperthermia-derived normalization descriptor while wound-specific biological validation is established [43]. Figure 1 summarizes these wound-interface heat-transfer and measurement considerations.

Table 2 groups thermal-dose concepts and practical dosing approaches relevant to local heating. Models developed for oncology hyperthermia, intact skin, and conventional surface warming provide useful terminology and safety concepts, with wound-specific validation required for application to chronic-wound protocols [66,70]. In this context, CEM43 is used as a hyperthermia-derived normalization metric, while lower-temperature wound-healing dose is characterized directly from measured thermal exposure.

In wound tissue, temperature influences linked vascular, metabolic, immune, and matrix pathways, but the evidence does not support a single universal therapeutic temperature. In healthy skin, local warming above approximately 37 °C produces progressively stronger cutaneous vasodilation, with a substantial nitric-oxide-dependent component near the higher end of commonly studied local-heating ranges [5,57]. At higher temperatures or longer exposures, physiological stimulation increasingly overlaps with cellular stress and thermal-injury mechanisms [73,74]. Table 3 therefore uses operational temperature contexts rather than fixed chronic-wound thresholds.

### 3.2. Determinants of Delivered Thermal Dose at the Wound Interface

At the wound interface, delivered dose is governed by surface geometry, hydration, dressing layers, contact pressure, and local perfusion. Moist or exudative tissue may conduct heat more readily than dry or callused margins. Movement and dressing compression can also change contact during treatment [76,77]. Flat, dry bench substrates therefore offer limited assurance of clinical performance [78]. Because hydrogel, occlusive, absorbent, and foam dressings differ in heat retention and moisture handling, each device-dressing combination should be tested under standardized wet, compressed, and clinically realistic conditions [79,80,81]. Heat penetration reflects a balance between conduction through the wound bed and convective heat removal by circulating blood. Conductive transfer depends mainly on tissue composition and water content, whereas convective transfer depends on local perfusion [82]. When perfusion rises during warming, convective cooling may limit further temperature rise in deeper layers and help stabilize oxygen tension. Studies of localized warming in vascular-compromised tissue support this feedback behavior and show that changes in blood flow affect local oxygen tension and temperature stability. In ischemic tissue, reduced perfusion weakens this protective effect. A surface temperature that appears acceptable may therefore create greater local overheating risk. Because most biophysical models do not incorporate these perfusion-dependent differences, their predictions may be unreliable in patients with significant vascular disease [70,72,82]. Reliable real-time temperature sensing is indispensable for safe dosing. Thin-film thermistors embedded in flexible heaters can give local measurements with good sensitivity and rapid response, but repeated bending, moisture ingress, and aging of encapsulation materials may cause drift [83,84]. In a closed-loop system, even a small sensor error can lead to unrecognized overheating or under-treatment because the controller responds to the reported temperature rather than the true tissue value. Few thermotherapy prototypes report calibration data, error margins, or response latency under realistic humidity, pressure, and movement. As a result, it is difficult to judge the reliability of temperature control or safety limits [85].

### 3.3. Implications for Wound-Specific Dosing and Device Design

Overall, the biological evidence provides a strong rationale for controlled, wound-specific investigation. Mild warming may improve perfusion, oxygenation, inflammatory signaling, heat-shock responses, and matrix remodeling, with supporting evidence spanning intact skin, acute models, and heterogeneous wound studies [41,86]. For device design and clinical protocols, the central requirement is to define the therapeutic window for each wound context. Future dosing studies should report achieved tissue-facing temperature, exposure duration, dressing configuration, moisture state, perfusion status, and clinically meaningful outcomes, including granulation, re-epithelialization, closure, pain, and tissue injury [41,43,51]. Figure 2 marks the intended transition from mild warming that may support repair to higher exposures associated with thermal stress and highlights the importance of integrating device setpoint with tissue risk, moisture, perfusion, and control reliability when defining safety margins [73,74].

For chronic wounds, thermotherapy therefore requires dose limits measured at the wound interface rather than inferred from nominal device settings. The following sections consider whether current device architectures, sensors, and control strategies can deliver such doses under realistic clinical conditions.

## 4. Device Architectures and Smart Thermotherapeutic Dressings

### 4.1. Heat-Delivery Principles and Device Design Space

Temperature-related wound technologies should be classified by mechanism and intended role before evidence is compared. Table 4 distinguishes direct therapeutic warming from multimodal photothermal systems and monitoring-only technologies, and separates direct human chronic-wound treatment evidence from engineering, preclinical, monitoring, and multimodal evidence. These distinctions reduce the risk of findings from one modality being generalized to technologies that differ in energy delivery, temperature context, therapeutic purpose, or biological co-interventions.

Figure 3 presents a qualitative engineering design map rather than an evidence ranking. The horizontal axis represents increasing conformability or substrate design flexibility, while the vertical axis represents increasing control complexity from open-loop operation toward sensor-integrated, adaptive, and connected control. Device positions are conceptual and indicate typical design tendencies rather than measured performance values [52,96,110].

### 4.2. Heater Platforms and Emerging Materials Systems

Heater platforms should be judged by the reliability of interface dosing rather than by heat generation alone. Conventional powered heating pads are familiar, simple, and already covered by established safety frameworks [15]. Their limited conformability can lead to uneven contact over irregular anatomy or layered dressings. Burns from body-heating devices have been documented, reinforcing the need for careful placement and monitoring [111]. Most conventional pads use thermostatic control or cut-offs rather than distributed interface sensing; advanced wearable systems can integrate real-time sensing and control [112]. FDA labeling guidance specifically warns against use over insensitive skin or in the presence of poor circulation [113]. Because existing standards mainly address dry blankets, pads, and mattresses, direct evidence for conventional heating systems over moist wound-dressing interfaces remains limited [16]. Liquid-metal microheaters use gallium-based conductive alloys, such as eutectic gallium-indium (EGaIn), embedded in soft elastomers through microchannels or patterned traces to produce deformable Joule heating [87]. These designs can maintain electrical continuity during bending and stretching while improving conformal contact. Better contact reduces interfacial air gaps that would otherwise impair heat transfer and may support more uniform surface heating than rigid traces on soft substrates [88]. Bench and in vivo demonstrations have shown rapid thermal ramping and relatively stable temperature fields under moderate strain, which is consistent with possible use on irregular skin and tissue surfaces. Leakage, channel fracture, and interface degradation remain important failure modes. Long-term stability during repeated handling and wet, variable-contact use is still insufficiently characterized [8,89,114]. Graphene and metallic thin-film heaters can provide thin, rapidly responsive, conformal heating layers for dressings or textiles, and they can be paired with sensing or wireless modules [90,91,115,116]. Their main translational risks include fatigue cracking, delamination, defect-related hot spots, and performance drift in moist, protein-rich wound environments. Across flexible-heater platforms, evidence remains limited for electrical safety, durability, and reproducible dosing under wound-like moisture, shear, and dressing-change conditions [92,93,94]. Hydrogel-integrated heaters provide close contact, high flexibility, and hydrated heat transfer across uneven wound surfaces. Hydrogels can also support drug-delivery functions, creating a route to combined thermal and pharmacologic treatment [97]. Swelling, drying, leachables, sterilization, and repeated handling can nevertheless change electrical output and mechanical integrity. These material changes should be treated as dose-control risks, not simply as fabrication problems [92,97]. Transient and bioresorbable platforms offer a route to short-duration devices that function during early repair and then resorb, avoiding device retrieval. An integrated flexible heater has been demonstrated in a biodegradable nanofibrous platform for on-demand drug delivery, although not as a clinically validated wound-warming system [98]. This approach is appealing for postoperative wounds and deep or difficult-to-access cavities, where removal may add risk. Most prototypes, however, still depend mainly on passive thermal behavior, including device geometry and intrinsic resistive properties, rather than closed-loop temperature sensing and feedback. During hydrolysis or corrosion, materials and interfaces change. Stiffness, electrical continuity, and contact conditions may shift, moving the spatial temperature profile away from the intended dose. The local and systemic tolerance of degradation by-products in inflamed, ischemic, or infection-prone tissue also remains incompletely characterized for many material classes. Regulatory translation must therefore justify both device performance over time and biological safety throughout degradation and resorption [99,100,101]. Photothermal wound dressings typically incorporate light-absorbing nanomaterials, such as Ti_3_C_2_T_x_ MXene, into hydrogel matrices so that external irradiation is converted into localized heat at the wound interface. MXene-enabled designs can heat rapidly and with spatial confinement. They are often combined with antimicrobial mechanisms, including photothermal killing, heat-triggered drug release, and MXene-associated antibacterial activity. In infected-wound animal models, the combination of bacterial suppression and thermal support has improved wound closure [102,103]. Recent preclinical platforms include a thermal-feedback hydrogel with self-limiting photothermal and thermoelectric functions, a long-acting mild-hyperthermia microneedle patch, and an alginate dressing with controlled heat release [117,118,119]. These systems broaden the engineering design space but do not constitute clinical efficacy evidence. Despite these preclinical results, nanomaterial safety remains a major translational concern. Depending on composition, surface terminations, oxidation state, and dose, MXenes and related two-dimensional particles may induce reactive-species-mediated stress responses, interact with blood and immune cells, or transform in ways that make in vivo behavior difficult to predict. Long-term retention and clearance are still incompletely defined for many MXene formulations. In chronic wounds, impaired vascular or lymphatic drainage may prolong residence time and alter local immune reactions. These uncertainties limit clinical viability even when laboratory and small-animal results are favorable [120,121].

Photothermal dressings are best interpreted as multimodal systems rather than as direct equivalents of normothermic warming. Combined photothermal-photodynamic therapy (PTT/PDT) couples light-to-heat conversion with photosensitizer-mediated reactive oxygen species generation, providing complementary antibacterial mechanisms relevant to infected wounds. Direct preclinical wound evidence includes a multifunctional bilayer hydrogel in an MRSA-infected rat wound model, in which PTT/PDT provided antibacterial control together with staged bioactive release supporting subsequent repair [122]. In such platforms, healing reflects the combined contribution of thermal, photodynamic, drug, material, and wound-microenvironment effects. Multifunctional antibacterial nanoplatforms can further combine PTT/PDT with chemodynamic effects [123], while photocatalytic materials such as graphitic carbon nitride provide ROS-mediated antibacterial activity and have been reviewed for biological applications including wound healing [124]. Other wound-oriented photothermal systems combine heating with drug release or material-mediated antibacterial activity [52,53,102,103]. Irradiance, exposure time, measured temperature, reactive-oxygen mechanisms, and co-interventions should therefore be reported as distinct components of the treatment description.

### 4.3. Smart Dressings, Sensing, and Closed-Loop Control

Smart dressings add sensing, feedback control, and workflow integration in an effort to narrow the gap between setpoint and delivered dose. Wireless hydrogel-based bioelectronic systems can combine microheaters, temperature sensors, and therapeutic reservoirs within conformal dressings, allowing programmable dosing and remote adjustment without disturbing the dressing [44,108,125]. The same integration introduces risks related to power management, packaging, cybersecurity, sensor-heater coupling, cost, and fault handling, especially during long wear in moist and mechanically variable wounds. Large-animal bioelectronic wound platforms provide a useful reference point for thermotherapy translation, particularly for packaging durability, wireless operation, and multi-day function in wet and mechanically dynamic wound environments. Baniya et al., for example, reported a wireless wound-healing actuator tested in a porcine model under conditions closer to clinical use. Although that system delivered electrical and drug-based therapy rather than heat, it illustrates the level of sealing, robustness, and workflow compatibility that future thermotherapeutic dressings will need to demonstrate [109].

Temperature sensing is the safety-critical component in closed-loop thermotherapy. Thin-film thermistors or resistance temperature detectors (RTDs) placed near the interface can provide fast local measurements, but bending, pressure variation, moisture ingress, and aging of the encapsulation can introduce calibration drift or signal artifacts [83,84,85]. Noncontact infrared thermography avoids direct contact, but exudate and dressing materials can alter emissivity and measurement conditions [104]. Wound-bed thermal imaging may nevertheless contribute to infection assessment [105]. Few prototypes quantify sensor accuracy, latency, and drift under the combined humidity, pressure, and movement conditions expected in chronic wound care. Integrated dressings should report sensor location, heater-sensor separation, encapsulation thickness, calibration method, measurement uncertainty, control logic, overshoot limits, and fault detection. Without this information, the displayed temperature should not be assumed to represent the true wound-interface temperature. Figure 4 shows how heater design, sensing position, electronics, exudate, contact pressure, and dressing materials together determine delivered dose and control reliability [92,110,126].

Overall, the field has moved away from rigid heaters and toward conformal, sensor-integrated, and multifunctional platforms. The clinical value of this added complexity will depend on whether it produces stable, measured, and safe interface temperatures during realistic use, not only during dry bench testing [127,128,129].

## 5. Regulatory, Safety, and Validation Considerations

### 5.1. Regulatory Pathways, Technical Standards, and Performance Validation

Regulatory classification depends on the product’s intended purpose, primary mode of action, energy source, software functions, duration and site of contact, and any combination with drugs or biologics; no single pathway covers all wound-thermotherapy platforms. In the United States, conventional powered heating pads are regulated under 21 CFR 890.5740 as Class II devices [15]. In the European Union, classification under the Medical Device Regulation depends on intended purpose and risk; active therapeutic devices that exchange energy are addressed under Rule 9, with class depending on whether energy delivery is potentially hazardous [130,131]. Accordingly, the technology archetypes in Table 5 are not assigned universal regulatory classes or submission pathways. The applicable pathway must be determined from the specific product configuration, intended purpose, mode of action, constituent functions, and jurisdiction. Flexible sensor-controlled heaters, externally activated photothermal materials, wireless closed-loop dressings, and heating-plus-drug systems can therefore require different regulatory analyses [130,131,132]. For eligible novel low- to moderate-risk devices without a legally marketed predicate, the U.S. FDA De Novo pathway can provide a classification route; whether it applies depends on the specific intended use and risk profile [133].

Horizontal standards remain relevant but must be matched to the device architecture. IEC 60601-1 addresses basic safety and essential performance for medical electrical equipment; IEC 60601-2-35 addresses heating devices using blankets, pads, or mattresses; IEC 60601-1-10 addresses physiologic closed-loop controllers; and IEC 60601-1-2 addresses electromagnetic compatibility [16,134,135,136]. ISO 14971 provides the risk-management framework [17]. Table 5 therefore separates established regulatory and standards frameworks that may apply to a given product from product-specific classification considerations and from the wound-interface validation criteria proposed by the authors for chronic-wound translation. To make these distinctions concrete, Table 5 is organized around representative device examples that span conventional warming, sensor-integrated heating, externally activated photothermal systems, connected closed-loop dressings, and heating combined with drug delivery.

Device safety depends on predictable thermal output, reliable insulation, and robust control during exposure to exudate, high humidity, compression, offloading, repeated handling, and variable contact pressure [17,83,84,85,140,141]. Under International Organization for Standardization (ISO) 14971, these conditions should be represented explicitly in hazard analysis, risk controls, verification testing, and residual-risk justification. Relevant failure modes include power instability, insulation breach, sensor drift, and control faults [17].

Many prototypes still demonstrate safety only under ideal laboratory conditions. Long-duration electrical and thermal performance in wet, mechanically stressed, dressing-compatible environments is rarely reported in a form that would support risk-based regulatory review [142]. Performance claims should be anchored to delivered thermal exposure. Premarket evidence should quantify heating rate, steady-state stability, peak temperature, spatial gradients, exposure duration, and the test conditions used to obtain those values. Because many wound-heating studies report only nominal setpoints or treatment schedules, standardized interface-dose reporting under moisture, pressure, and dressing variation is important for regulatory credibility as well as clinical interpretation [143,144,145].

### 5.2. Human Factors, Usability, and Workflow-Related Risks

Human factors engineering focuses on how clinicians and patients interact with devices and is an expected part of US and EU medical-device development when safe use must be demonstrated under intended conditions. Evaluations typically address interface clarity, ease of placement, alarm visibility, and the adequacy of labeling and instructions because these features influence whether a device can be used safely and consistently. For chronic-wound thermotherapy, human factors assessment also points to the need for simple temperature controls, clear treatment-progress indicators, and explicit warnings for high-risk conditions such as ischemia or impaired sensation [113,139]. Published usability testing remains limited for many thermotherapeutic systems, especially in settings that resemble clinical workflows or home-based care. This matters because foreseeable use errors can materially change delivered thermal exposure. IEC 62366-1 requires a usability-engineering process focused on safety-related use errors [146]. Heating-pad recalls involving overheating, electrical faults, and burn injuries illustrate the consequences of control or manufacturing failures [147].

### 5.3. Cybersecurity and Data Governance

As thermotherapeutic devices incorporate wireless sensing or remote monitoring, cybersecurity becomes part of the regulatory assessment of connected medical devices. Remote temperature monitoring in high-risk diabetic foot care illustrates why secure communication and protected data transmission matter when streamed measurements may influence clinical decisions [106]. Current regulatory expectations emphasize documented controls, including authentication, encryption, secure update mechanisms, and lifecycle processes that reduce the likelihood of unauthorized changes to device parameters. Many wireless thermotherapy prototypes still do not report cybersecurity assessment methods or validation evidence. Connected systems therefore require cybersecurity risk management across the product life cycle and evidence appropriate to premarket and postmarket oversight [137,148,149].

### 5.4. Quality Management and Implications for Developers

Manufacturers must meet quality-system requirements that formalize design controls, risk documentation, verification and validation, and post-market quality activities across the device life cycle. In the United States, the FDA Quality Management System Regulation aligns quality expectations with ISO 13485:2016 by incorporation by reference [150,151,152]. This reinforces the need for documented processes that support consistent design, testing, and change control. For thermotherapeutic devices, performance testing should reflect foreseeable use conditions, including moisture exposure, repeated handling, and interactions with dressing materials that can alter heat transfer and electrical integrity. Clear documentation of design assumptions, test criteria, and performance limits is necessary for regulatory acceptance and for judging whether bench evidence is transferable to clinical use. Many academic prototypes are not developed within a regulatory-grade quality system, and missing verification evidence can become a primary barrier to translation even when the basic device function appears promising. These expectations directly affect whether a promising heater can become a reproducible wound-care device. A wound-relevant verification framework is still needed to connect electrical safety, heat uniformity, sensing reliability, software behavior, human factors, and post-market surveillance for flexible and connected systems [130,134,151]. The clinical evidence should therefore be interpreted within this regulatory and validation context.

## 6. Clinical Evidence and Translational Implications

### 6.1. Overview of Clinical Studies and Wound Types

Direct human treatment evidence for established chronic wounds is concentrated in historical radiant or noncontact normothermic wound therapy (NNWT) studies, together with a limited number of wIRA and other local-heat reports. These studies provide the clinical foundation for the present synthesis, while contemporary flexible, wireless, and photothermal platforms are considered according to their own engineering and preclinical evidence. Schumann et al. [12] is included as modality-specific wIRA + VIS evidence and interpreted in relation to the combined intervention. Scott et al. [13] is retained as prevention-related contextual evidence because it evaluated intraoperative forced-air warming for prevention of postoperative pressure ulcers. Galdino-Junior et al. [39] is retained as supportive/contextual clinical evidence because it evaluated warmed saline during dressing changes. The systematic review by Yue et al. [14] screened 139 records and found no RCT meeting its prespecified eligibility criteria. Of the three full-text reports assessed, one was considered nonrandomized, one evaluated warming together with co-interventions, and one reported use of a random-number table with insufficient methodological detail to establish randomization. The present synthesis therefore distinguishes the design terminology reported in individual primary publications from eligibility under Yue et al.’s later systematic-review criteria. Table 6 and Supplementary Tables S2 and S3 use design terminology supported by the primary reports.

The clinical relevance of local warming depends on wound context and should not be inferred from the presence of treatment evidence alone. The available direct human evidence is concentrated primarily in venous, pressure, and diabetes-related wounds, whereas evidence for arterial/ischemic and mixed-etiology wounds is more limited. Differences in evidence availability should be considered separately from wound-specific factors that may influence the safety and reproducibility of thermal intervention, including impaired perfusion, neuropathy or sensory loss, infection, mechanical loading, and interaction with standard etiologic care. Postoperative warming evidence considered in this review is contextual and prevention-related rather than evidence of thermotherapy for established chronic wounds. Accordingly, the present synthesis does not assign numerical evidence or safety rankings to individual wound types.

Supplementary Table S2 and Table 6 summarize the core direct-treatment reports using information directly supported by the accessible publication record. Supplementary Table S2 reports total sample size and treatment/control group sizes where available, distinguishes enrolled or randomized populations from analyzed/completer populations where relevant, and identifies whether the reported unit is the patient or the wound. For studies in which a participant contributed more than one wound, patient and wound counts are reported separately and the analytical level used in the primary study is identified. The table also summarizes assignment/comparator, baseline context, thermal intervention, target or device setpoint, achieved or measured temperature, measurement site, follow-up, attrition, and adverse-event reporting. Target/setpoint and measured wound or periwound temperature are reported as separate quantities. Follow-up is presented as the documented observation period or stopping rule, and attrition is reported by distinguishing enrolled or randomized populations from analyzed or completer populations where these differ. Adverse-event reporting is recorded according to the wording of the primary publication. Table 6 presents study-specific quantitative outcome signals together with the corresponding thermal-dose and methodological context, including healing rate, proportion healed, time to prespecified wound-area thresholds, wound-area or volume reduction, recurrence, pain scores, and associated significance values where reported. Supplementary Table S3 provides a study-level qualitative risk-of-bias appraisal based on assignment, attrition and unit of analysis, co-interventions or blinding, and thermal-dose measurement. Across these tables, only values and study characteristics supported by the accessible source record are reported, and unavailable items are identified explicitly.

### 6.2. Thermal Dosing, Temperature Control, and Microcirculatory Outcomes

The main gap in clinical reporting is characterization of the delivered thermal exposure. Most studies describe an intended setpoint, nominal device behavior, or treatment schedule instead of continuous interface temperature, spatial uniformity, peak temperature, recovery after dressing changes, and cumulative exposure. Without these data, outcomes cannot be linked confidently to biological effect, device performance, or application variability [41,57]. Microcirculatory measures should be interpreted as mechanistic intermediates, not as substitutes for healing endpoints. Local warming can increase superficial flow and oxygenation, but future trials should test whether deeper hypoxic wound regions receive meaningful oxygen delivery. This will require dose logs paired with prespecified perfusion or oxygenation measures and closure-related outcomes [56,153]. Near-infrared spectroscopy is another noninvasive approach for characterizing wound-tissue oxygenation and perfusion and may complement, rather than replace, healing endpoints [154]. Patient-reported outcomes and safety events should be collected prospectively, including heat-related comfort, pain during dressing changes, adherence, maceration, burns, worsening infection, and device-related pressure injury [39,41]. Because heat-pain profiles vary even among pain-free volunteers, thermal discomfort should be measured directly rather than inferred from device temperature alone [155].

### 6.3. Methodological Quality and Outcome Measures

Supplementary Table S3 shows that the principal limitations are small samples, incomplete allocation or attrition reporting, wound-versus-patient unit issues, lack of blinding in several reports, co-interventions, and incomplete thermal dosimetry. Yue et al. screened 139 records and assessed three full-text reports, none of which met the review’s prespecified inclusion criteria [14]. Because that review does not provide a study-by-study crosswalk for the historical NNWT reports summarized here, the present review does not retrospectively reclassify those studies. Randomized-design terminology therefore reports the description supported by the primary publication and is kept distinct from eligibility under the systematic-review criteria of Yue et al. [14]. Future protocols should follow SPIRIT guidance, completed randomized trials should follow CONSORT, and device-based thermotherapy should be described with TIDieR-level detail, including temperature-control logic, sensor placement, dressing stack, co-interventions, adherence, and adverse-event capture [156,157,158].

### 6.4. Wound Settings Most Likely to Benefit and Major Risk Groups

Current evidence positions thermotherapy as an investigational adjunct rather than routine care. The most plausible candidate wounds are those with impaired but recoverable perfusion, delayed granulation, persistent inflammation, and preserved capacity for microvascular response. Venous leg ulcers and pressure injuries provide the clearest human treatment signals to date, although the evidence base remains limited and methodologically heterogeneous [41].

Severe ischemia, neuro-ischemic disease, diabetic neuropathy, active infection, and mixed-etiology ulcers remain high-risk settings. Poor perfusion reduces heat removal, sensory loss may mask overheating, peripheral arterial disease can blunt vascular redistribution, and infection may increase metabolic demand in oxygen-limited tissue. Until temperature limits, exposure times, stopping rules, and monitoring requirements are defined, unsupervised use in these groups is not justified [159,160,161,162,163,164].

### 6.5. Thermotherapy in Combination with Standard and Adjunctive Wound Care

Established chronic-wound care is etiology-specific and can include compression for venous disease, offloading for diabetes-related foot disease, pressure-injury management, debridement, infection management, moisture balance, and NPWT in selected wounds [159,165,166,167,168,169,170]. NPWT has randomized clinical evidence in diabetic foot ulcers and has also been studied with wound thermometry; these data concern NPWT itself and do not establish benefit from adding heat [171,172,173]. Thermotherapy should therefore be studied as an adjunct within established care pathways rather than as an isolated intervention. Potential interactions with NPWT, compression, offloading, and antimicrobial strategies require direct testing because each can alter moisture, contact, perfusion, or local thermal distribution. Table 7 summarizes possible roles, major cautions, evidence position, and knowledge gaps by wound context.

Compression therapy is central to venous leg ulcer care, and superficial warming has shown potential in this setting. If heating devices are placed beneath or near compression systems, pressure may improve contact and heat transfer. At the same time, trapped moisture and an altered microclimate may increase local thermal exposure. The interaction could strengthen treatment effects, but it could also raise risk, particularly in patients with mixed arterial disease [164]. Existing studies address wound warming [14], compression-related pressure and moisture effects [174], or compression sensing [175] separately; they do not establish temperature distribution, vascular response, or safety limits for combined warming and compression. Debridement removes devitalized tissue and bacterial burden and may create a short interval in which improved perfusion could support early granulation. Applying heat after debridement is therefore plausible, but it has not been tested in controlled clinical studies. Dressing changes also alter moisture, temperature, and interface contact, all of which influence heat transfer. Future protocols should specify the timing of thermotherapy relative to debridement, cleansing, dressing changes, compression, and offloading so that variability in delivered dose is reduced [50,176,177]. Chronic wounds often carry a high bacterial burden. Warming could theoretically support host clearance by improving perfusion, or it could be paired with temperature-responsive antimicrobial dressings and controlled drug release. These combined strategies remain largely preclinical. Clinical studies are needed to determine whether warming improves antimicrobial efficacy, changes bacterial growth, alters exudate dynamics, or creates safety concerns in infected or heavily colonized wounds [10,178,179].

### 6.6. Care Models, Home-Based Use, and Remote Monitoring

Remote temperature monitoring can detect early tissue changes in high-risk diabetic feet and has been associated with reduced ulcer formation and amputation in real-world programs [107]. These findings support temperature-informed care, but they should not be interpreted as evidence for therapeutic heating. In home-based thermotherapy, remote monitoring may eventually support supervised dosing, adherence tracking, and alerts. Home wearable and digital foot-care systems have been demonstrated [180,181], but adherence remains a documented limitation in remote temperature monitoring [182,183]. Data quality, cybersecurity, sensor stability, usability, and safe long-wear performance still require direct evaluation [148,184]. Implementation will depend on workflow as much as device design. Qualitative research on virtual chronic-wound care similarly highlights the importance of patient education, multidimensional care needs, and implementation support [185]. Clinicians, caregivers, and patients need simple controls, clear alarms, placement guidance, instructions on dressing compatibility, and explicit responses to fault conditions. Correct use should be verified in representative-user studies because placement or operating errors can produce variable dosing and inconsistent outcomes [139,186]. Overall, the clinical evidence supports controlled investigation rather than routine adoption. In research settings, thermotherapy should remain an adjunct to standard care in carefully selected wounds, with particular caution in poor perfusion, reduced sensation, active infection, or mixed etiology [159,168].

## 7. Future Directions and Roadmap

### 7.1. Mechanistic Gaps and Wound-Specific Dose Frameworks

Future mechanistic studies should move beyond intact-skin physiology and simplified cell or bench models. Experimental systems need to reproduce exudate, dressing layers, heterogeneous tissue, impaired perfusion, bacterial burden, and mechanical loading. These variables determine both heat distribution and biological response. Priority assessments include perfusion, oxygenation, inflammatory mediators, heat-shock responses, matrix remodeling, and, where feasible, direct measurements in human chronic-wound tissue [55,75,187]. CEM43 and related metrics remain useful for comparison, but they should not be used as standalone dose definitions for chronic wounds. Wound-specific models should include geometry, moisture, dressing configuration, tissue heterogeneity, and perfusion-dependent heat removal. General hyperthermia modeling confirms that perfusion can materially change heat retention [188]. Wound-specific models should therefore be validated against temperature maps, perfusion measures, and clinical outcomes [42]. Dedicated safety studies are especially needed in patients with poor perfusion, neuropathy, and mixed etiologies. These patients may need perfusion support but may also have reduced ability to dissipate or perceive harmful heat. Conservative ceilings, exposure durations, stopping rules, and monitoring requirements should be defined before unsupervised or home-based use is considered [189,190].

### 7.2. Translational Readiness and Engineering Constraints

Because thermotherapeutic platforms differ in mechanism and maturity, translational assessment is most informative when it identifies the failure mode that most directly threatens wound-interface dose validity. Table 8 therefore summarizes one priority failure mode for each emerging heater class and the evidence needed to address it. These priorities are deliberately narrower than the broader platform classification in Table 4 and focus on whether the intended thermal exposure remains stable under moisture, deformation, pressure, optical attenuation, and material change [45,46,47,53,114,116,191,192,193,194,195,196,197,198,199].

### 7.3. Standardization of Endpoints, Trial Design, and Combined-Modality Protocols

Future trials should make thermal interventions reproducible. Existing core-outcome and outcome-classification work in complex wounds, venous leg ulcers, and pressure-ulcer research underscores the need to harmonize wound outcomes, while chronic wounds also impose important quality-of-life burdens [200,201,202,203]. Protocols should follow SPIRIT, completed randomized trials should follow CONSORT, and device-based interventions should be described using TIDieR. Reports should include sensor placement, control logic, dressing stack, treatment schedule, co-interventions, adherence, and adverse-event capture [156,157,158,201]. A minimum reporting set should include wound diagnosis and etiology; perfusion and sensory status; target and measured interface temperature; sensor type, location, calibration, response time, and uncertainty; duration, frequency, adherence, dressing configuration, moisture condition, co-interventions, spatial uniformity, peak temperature, adverse thermal events, wound-area method, and patient-relevant outcomes [156,157,158]. Wound-area measurement methods should be validated and described explicitly [204,205]. Protocols that combine thermotherapy with compression, NPWT, debridement, antimicrobial dressings, or bacterial-burden management should test synergy rather than assume it. They should specify how timing and co-interventions affect perfusion, drug delivery, moisture balance, contact pressure, exudate handling, sensor accuracy, and thermal distribution [156,157,201].

### 7.4. Regulatory Evolution, Digital Health, and Intelligent Control

Physical wound-interface durability and platform-specific failure modes are summarized in Table 8. For connected and adaptive thermotherapy systems, additional translational requirements arise from sensing, software, communications, cybersecurity, closed-loop behavior, and long-term data quality. Remote monitoring and automatic control may eventually support supervised home thermotherapy, adherence tracking, and alerts. Cybersecurity and closed-loop control requirements are addressed by FDA guidance [148,206], while foot-monitoring studies identify adherence and implementation limitations [107,180,183]. Sensor accuracy, data quality, privacy, and long-term performance in the home still require direct evaluation. Adaptive wound bioelectronic platforms provide useful design analogies for thermotherapy because they combine distributed sensing, wireless communication, and closed-loop control. Systems such as a-Heal show how diagnostics and therapy may eventually be integrated in advanced wound devices. They should be interpreted as translational analogies rather than direct evidence for thermal wound treatment [207]. Adaptive algorithms could personalize heating according to perfusion, moisture, temperature history, or healing trajectory. They also introduce verification and regulatory challenges. Recent FDA guidance on artificial intelligence (AI)-enabled device software and predetermined change control plans is relevant to this direction [208,209,210]. Before intelligent heat-control systems can be used routinely, developers will need hardware-in-the-loop testing, fault handling, overshoot limits, drift detection, cybersecurity controls, and monitored feasibility studies under realistic wound conditions [206,207,208,209,210].

### 7.5. Integrated Roadmap Priorities

As summarized in Figure 5, progress toward routine wound-care use will require five linked advances: wound-specific dose definition, validation under realistic interface conditions, standardized reporting, clinically informative trials, and translation pathways that address usability, manufacturing, cybersecurity, safety, and regulation [137,139,207].

Table 9 summarizes priority research questions across chronic-wound mechanisms, dose modeling, device durability, high-risk patient groups, combined treatments, hybrid-device standards, remote monitoring, and adaptive control. For each area, it identifies suggested methods, core measures, and translational relevance so that future studies are easier to compare and more useful for device development and clinical decision-making.

In practical terms, priority should be given to wound-relevant mechanistic studies, moist and pressurized interface validation, early safety studies in high-risk limbs, controlled combination protocols, and regulatory-grade evidence for connected or adaptive devices.

## 8. Conclusions

Thermotherapy is biologically plausible for selected chronic wounds, but it is not yet a practice-defining adjunct. This review treats local warming as a wound-interface, dose-controlled device intervention and separates modalities that differ substantially in energy delivery and biological co-effects. Clinical credibility depends on whether a defined exposure can be specified, delivered, measured, controlled, and justified under the biological, mechanical, and workflow conditions of chronic-wound care. Evidence from historical NNWT or wIRA should not be transferred directly to Joule-heated, photothermal, wireless, or multimodal platforms without direct supporting data.

Flexible heaters, hydrogel systems, photothermal materials, bioresorbable devices, and sensor-guided dressings broaden the technical options for local warming, with interface-level evidence providing the basis for translation. For lower-temperature wound warming, complete measured time–temperature profiles, peak temperature, spatial variation, measurement location, and sensor uncertainty provide the most informative dose description, while CEM43 can serve as a secondary normalization metric during wound-specific biological validation. Future work should emphasize wound-specific dosing frameworks, realistic interface validation, standardized clinical reporting and risk-of-bias assessment, and controlled evaluation alongside established wound-care practices. Together, these steps provide a clear evidence pathway toward safe, reproducible, and clinically credible thermotherapy modalities.

## Figures and Tables

**Figure 1:**
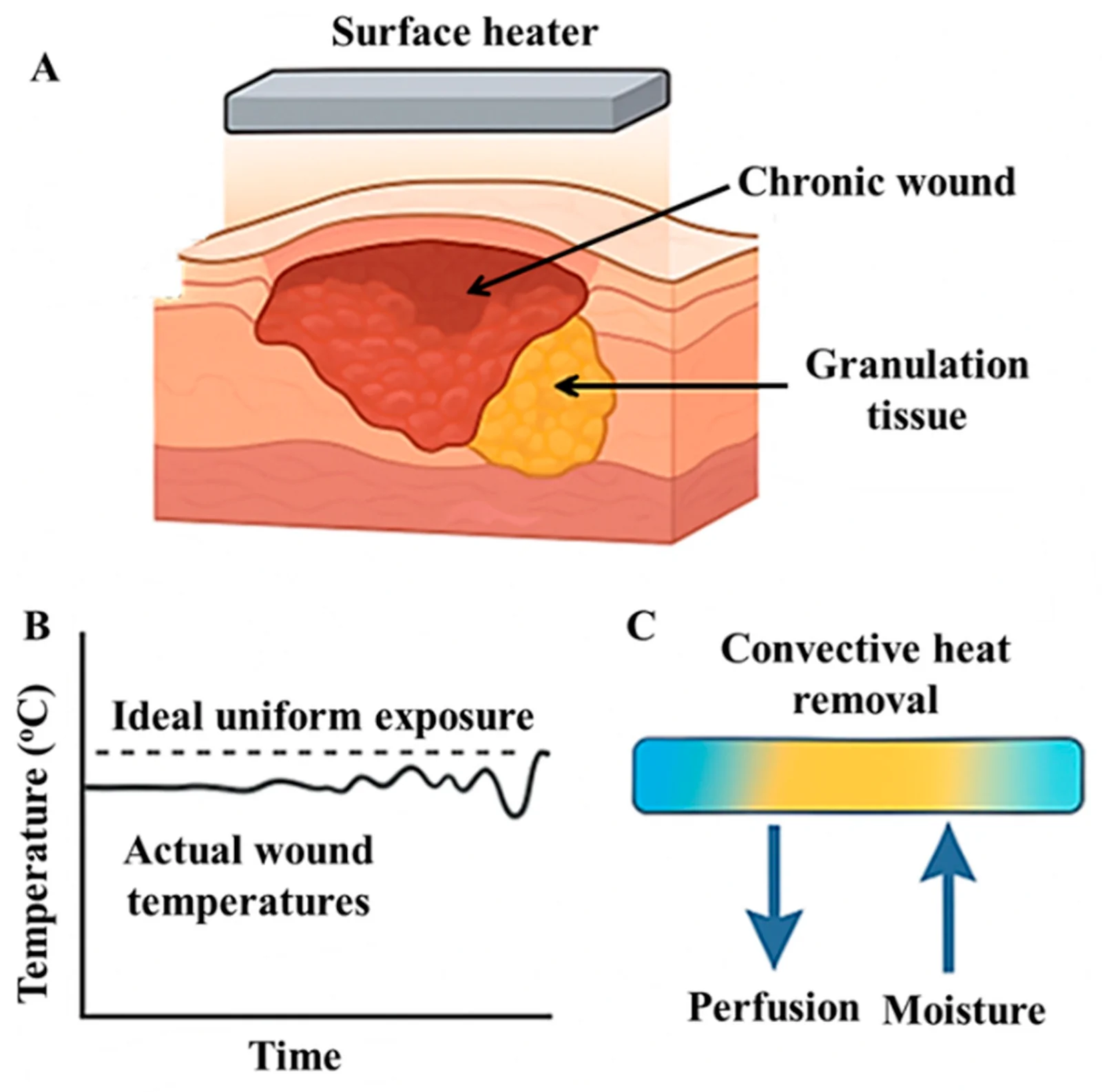
Wound-interface heat transfer: (**A**) surface heating; (**B**) idealized versus variable interface temperatures; (**C**) perfusion- and moisture-mediated heat removal.

**Figure 2:**
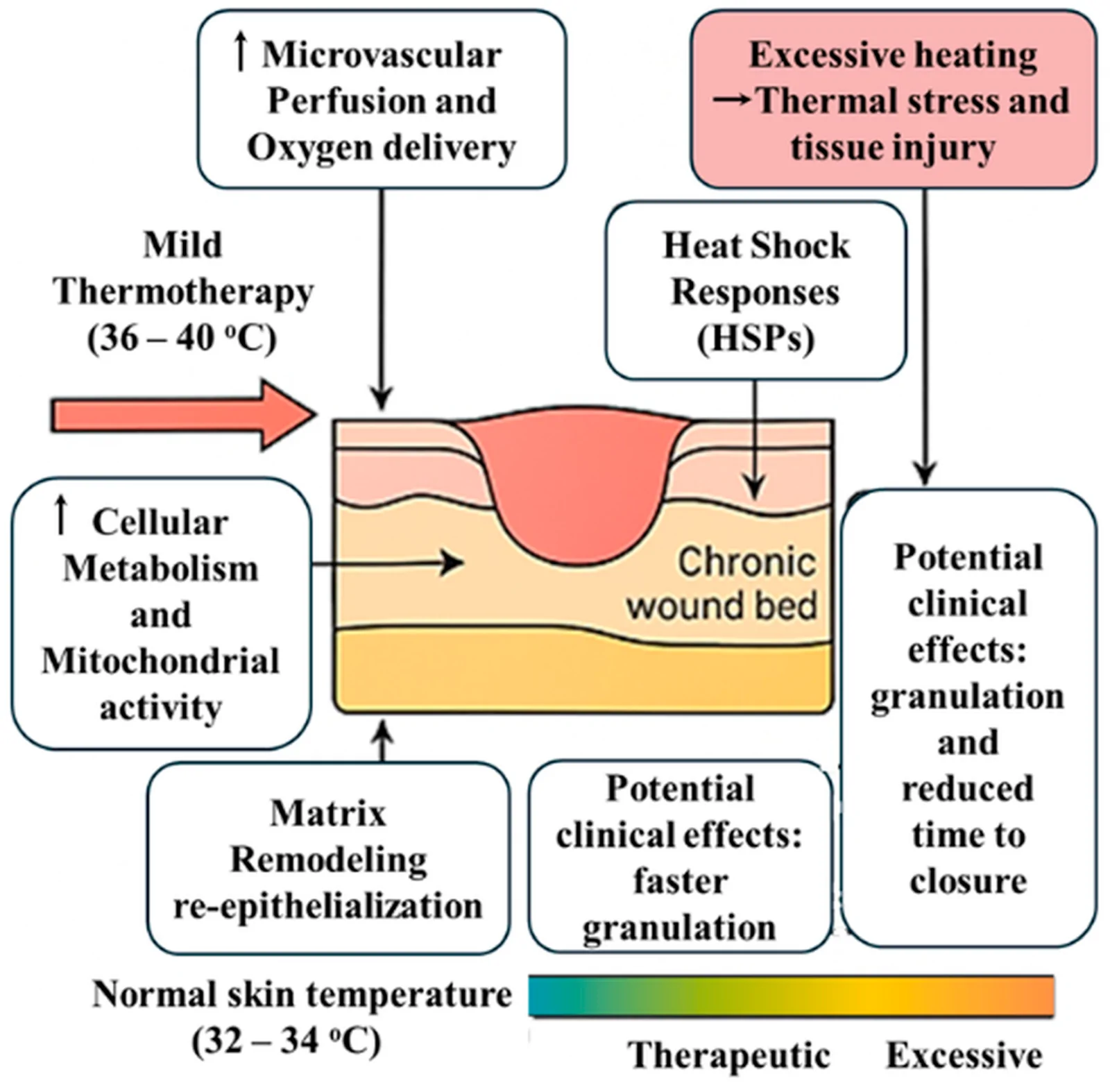
Mechanistic pathways linking controlled local warming to chronic-wound healing and thermal risk. The 36–40 °C label denotes the operational mild-thermotherapy range used in this review, not a validated universal chronic-wound therapeutic window. Responses depend on exposure duration, modality, tissue perfusion, and measurement location. Arrows indicate conceptual relationships among thermal exposure, biological responses, and wound outcomes. The green-to-orange gradient indicates a qualitative transition from therapeutic to excessive thermal exposure and does not represent a quantitative scale. Abbreviation: HSP, heat shock protein.

**Figure 3:**
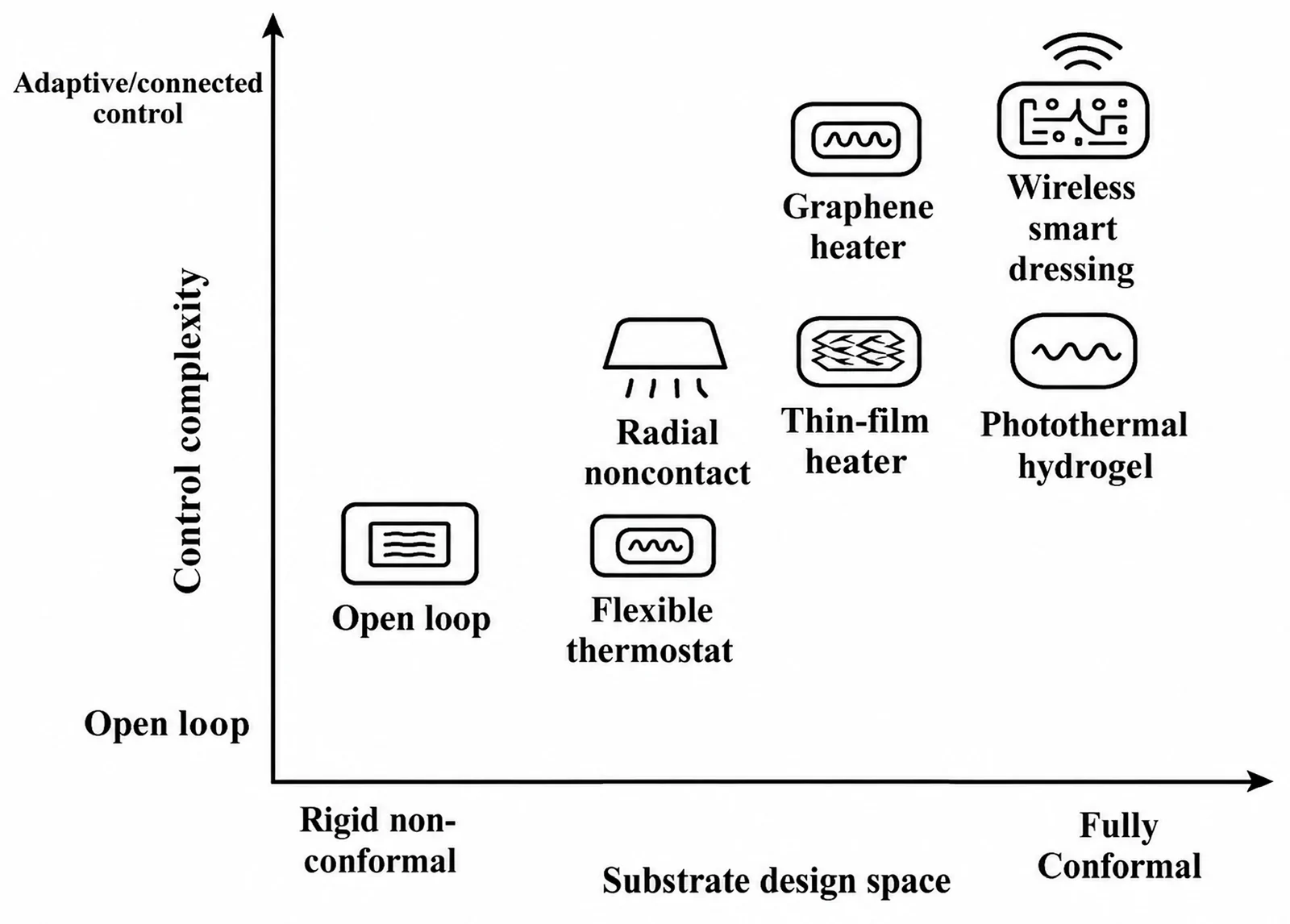
Qualitative engineering design space for thermotherapeutic devices. The horizontal axis represents increasing conformability or substrate flexibility, and the vertical axis increasing control complexity from open-loop to adaptive or connected control. Symbol positions are conceptual and do not indicate quantitative performance, clinical maturity, or comparative efficacy.

**Figure 4:**
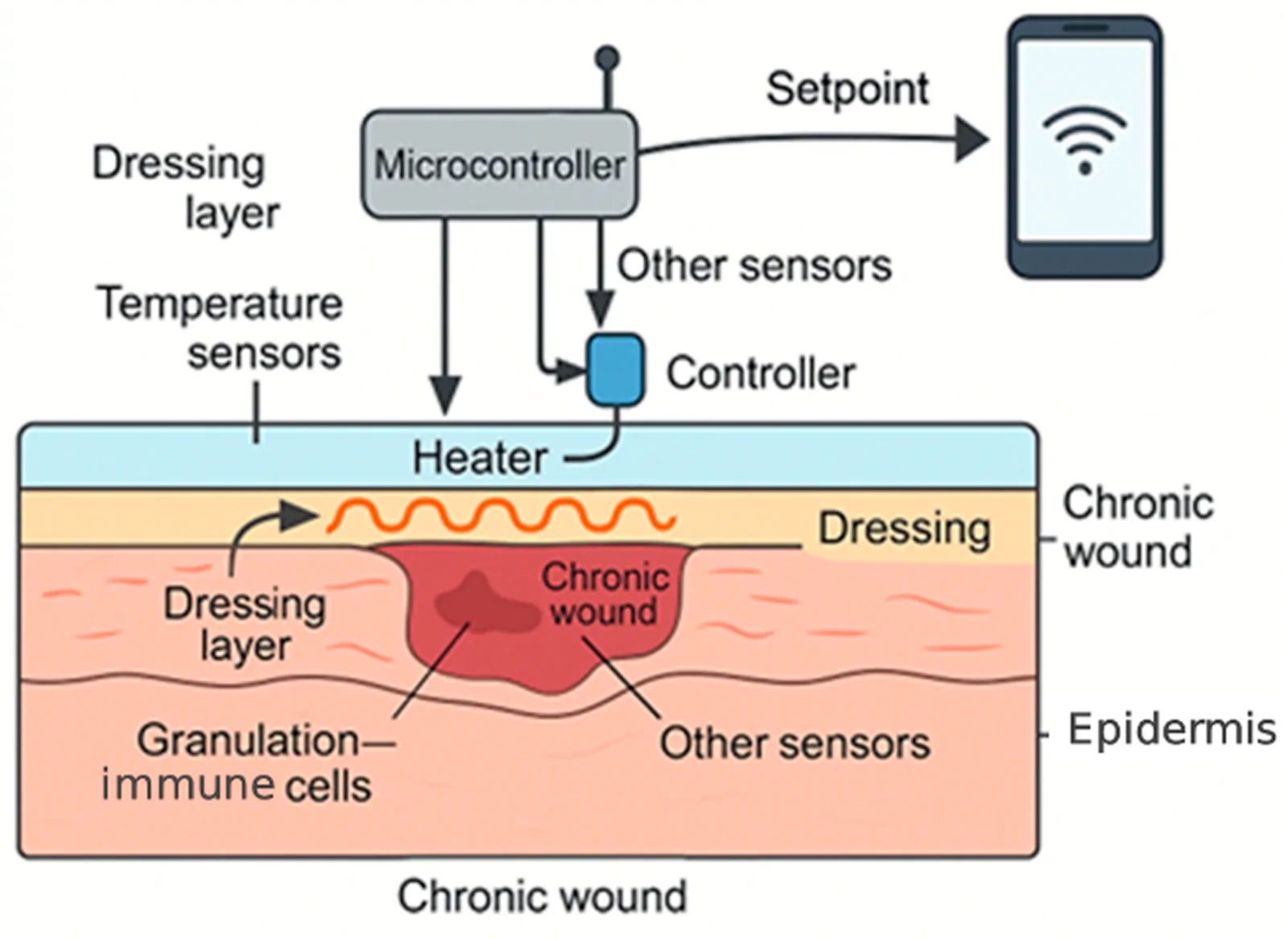
Conceptual architecture of a sensor-guided thermotherapeutic dressing with feedback control. Temperature and auxiliary sensors inform a controller that adjusts heater output relative to the prescribed setpoint; sensor position is important because the measured temperature may not represent the actual wound-interface temperature.

**Figure 5:**
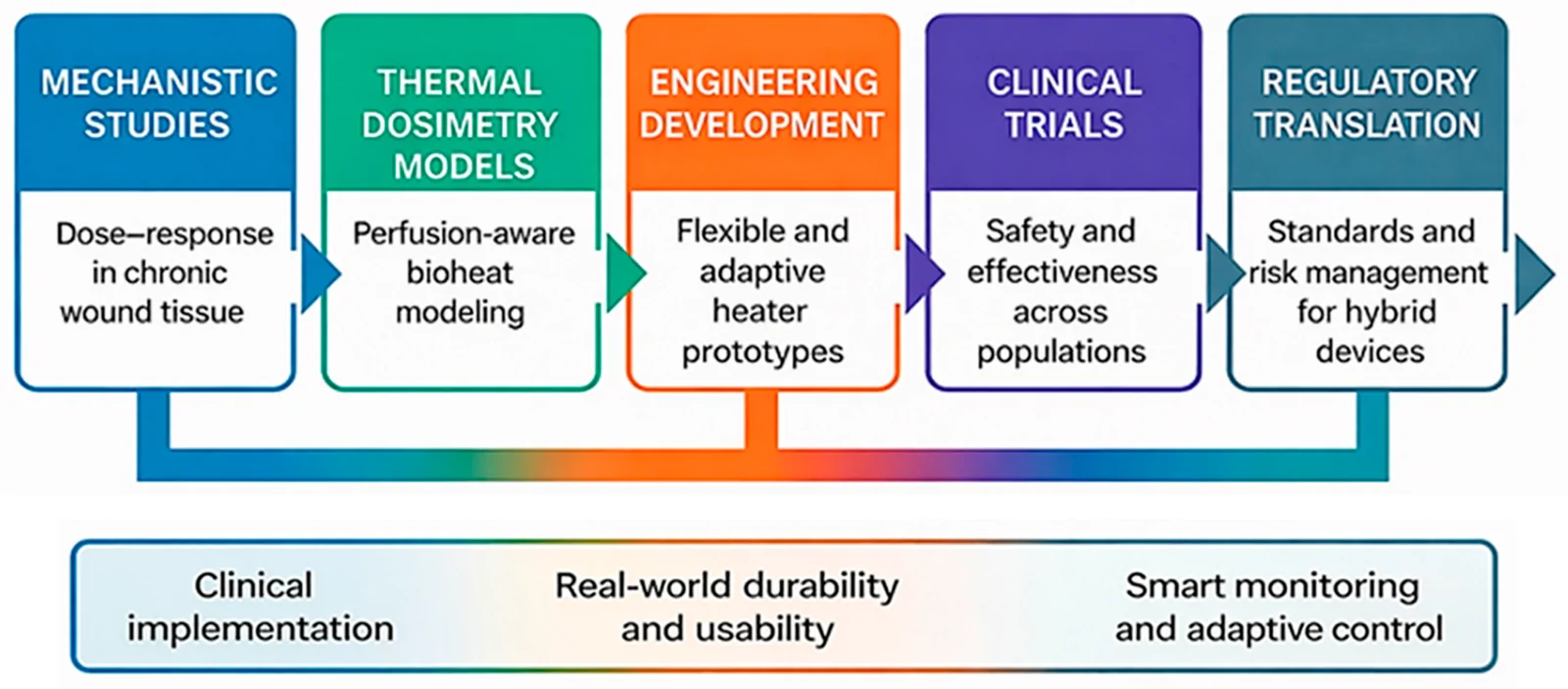
Translational roadmap for thermotherapeutic wound devices. The upper sequence summarizes five development priorities from mechanistic dose–response studies to regulatory translation; the lower band highlights cross-cutting priorities for implementation, durability, usability, and adaptive monitoring. Arrows indicate a conceptual progression rather than a validated linear pathway.

**Table 1:** Focused clinical-evidence classification and report-specific disposition.

Classification	Report	Report-Specific Rationale
**Core direct-treatment** **synthesis**	Robinson and Santilli, 1998 [18]	Established chronic venous stasis ulcers; local noncontact radiant warming evaluated as treatment with healing-related outcomes.
	Santilli et al., 1999 [19]	Established chronic venous stasis ulcers; local radiant warming evaluated as treatment with wound-healing outcomes.
	Cherry and Wilson, 1999 [20]	Established venous ulcers; local warming to 38 °C used therapeutically with healing follow-up.
	Price et al., 2000 [21]	Established Stage III–IV pressure ulcers; radiant warming compared with standard treatment and healing outcomes reported.
	Kloth et al., 2000 [22]	Established Stage III–IV pressure ulcers; semiocclusive heated dressing at 38 °C evaluated against standard care.
	Biland and Barras, 2001 [23]	Chronic venous ulcers treated with wIRA; primary report is brief, so detailed study-level interpretation remains limited.
	Whitney et al., 2001 [24]	Established Stage III–IV pressure ulcers; NNWT at 38 °C evaluated in a prospective randomized trial, as described in the primary report.
	McCulloch and Knight, 2002 [25]	Neuropathic diabetic foot ulcers; noncontact radiant warming plus offloading compared with offloading alone.
	Kloth et al., 2002 [26]	Established Stage III–IV pressure ulcers; NNWT evaluated in a prospective randomized controlled trial, as described in the primary report.
	Alvarez et al., 2003 [27]	Neuropathic diabetic foot ulcers; interim randomized controlled report of NNWT versus standard care, as described in the primary publication.
	Karr, 2003 [28]	Established lower-extremity wounds associated with managed osteomyelitis; NNWT evaluated with healing outcomes.
	Thomas et al., 2005 [29]	Established Stage III–IV pressure ulcers; radiant heat dressing evaluated in a randomized comparative pilot, as described in the primary report.
	Alvarez et al., 2006 [30]	Diabetic neuropathic foot ulcers; PubMed-indexed randomized controlled report of NNWT. Report-level counting does not assume independence from other publications by the same investigator group.
	Mercer et al., 2008 [31]	Recalcitrant chronic venous stasis ulcers; wIRA + VIS used therapeutically with wound-healing and thermographic outcomes; comparative interpretation is limited by the de facto cohort design.
	Petrofsky et al., 2010 [32]	Nonhealing diabetic foot ulcers; a local dry-heat-only arm at 37 °C permits direct observation of a thermal treatment arm, although there was no no-heat comparator.
	Schumann et al., 2011 [12]	Nonhealing chronic venous stasis ulcers; randomized wIRA + VIS versus VIS study, as described in the primary report. The intervention is modality-specific and includes thermal and nonthermal/nonthermic effects.
**Supportive/contextual** **clinical evidence**	Hoffmann, 1994 [33]	Chronic-ulcer case evidence involving wIRA-induced localized hyperthermia and hyperbaric oxygen; the thermal contribution cannot be isolated.
	Ellis et al., 2003 [34]	Full-thickness pressure sores treated with radiant warming, but the principal study endpoint was MRSA eradication rather than wound-healing efficacy.
	Lawson and Petrofsky, 2007 [35]	Chronic wounds studied with electrical stimulation in a warm-room context; the contribution of heat cannot be separated from the active electrical intervention.
	Petrofsky et al., 2007 [36]	Chronic diabetic wounds treated with electrical stimulation plus either local or global heat; thermal effects cannot be interpreted as an independent wound-healing intervention.
	Petrofsky et al., 2007 [37]	Human wound/skin blood-flow study examining environmental temperature effects on the response to electrical stimulation; supports physiological context rather than stand-alone thermotherapy efficacy.
	Suh et al., 2009 [38]	Chronic ulcers treated with local heat at 37 °C together with electrical stimulation; no heat-only comparator, so the thermal contribution cannot be isolated.
	Galdino-Junior et al., 2024 [39]	Evaluated warmed saline during dressing changes rather than a sustained local heating intervention intended to influence wound healing.
**Contextual prevention evidence;** **not included in the core** **direct-treatment synthesis**	Scott et al., 2001 [13]	Evaluated intraoperative forced-air warming for prevention of postoperative pressure ulcers rather than treatment of an established chronic wound.
**Not included in the core** **direct-treatment synthesis**	Suh et al., 2009 [40]	Evaluated an electrical-stimulation electrode system without a therapeutic thermal intervention; not thermotherapy evidence.

Abbreviations: MRSA, methicillin-resistant Staphylococcus aureus; NNWT, noncontact normothermic wound therapy; VIS, visible light; wIRA, water-filtered infrared A.

**Table 2:** Thermal dose frameworks and practical dosing approaches relevant to chronic wound thermotherapy.

Framework/Approach	Typical Basis	Original Context	Main Assumption	Relevance to Chronic Wounds	Main Limitation
CEM43 [42,43]	Time–temperature history converted to equivalent minutes at 43 °C	Oncology hyperthermia	Tissue response can be approximated by a common thermal-dose relationship	Provides a common hyperthermia normalization language; may be reported secondarily when the measurement site is explicit.	Biological meaning at approximately 36–40 °C wound warming is unvalidated; results depend strongly on measured temperature location and time history.
Fixed temperature and time [19,26,27]	Prespecified surface temperature or normothermic warming schedule	Pilot wound warming studies; practical clinical protocols	Delivered temperature is reasonably stable across the treatment field	Simple to implement and compare operationally	Actual interface temperature and spatial uniformity are often unknown
Perfusion/O_2_ targets [70,71,72]	Heating adjusted according to TcPO_2_, perfusion response, or related physiological surrogates	Microcirculation and vascular assessment studies	Surrogate physiological improvement reflects useful local heating	Potentially relevant in ischemia-prone wounds	Requires monitoring infrastructure and remains weakly validated as a dosing strategy
Max temperature limit [73,74]	Upper temperature bounds intended to reduce tissue injury risk	Thermal safety literature; burn prevention	A conservative ceiling can reduce the likelihood of overheating	Important for safety framing, especially in high-risk wounds	Thresholds are largely extrapolated from intact skin and may not translate directly to ischemic or neuropathic tissue
Wound empirical protocols [18,19,26,27]	Device-specific treatment schedules used in early wound studies	Early clinical wound warming literature	Similar devices and wound settings will respond similarly	Useful as historical starting points	Poor standardization, incomplete dose reporting, and limited generalizability

Abbreviations: CEM43, cumulative equivalent minutes at 43 °C; O_2_, oxygen; TcPO_2_, transcutaneous oxygen tension.

**Table 3:** Operational temperature contexts and reported physiological or biological responses relevant to wound healing.

**Operational Temperature Context**	**Reported Physiological/Biological Response**	**Potential Wound Relevance**	**Evidence Limitation/Interpretation**
Approximately 32–34 °C: resting skin-surface reference, not a treatment target	Surface temperature varies with anatomical site, perfusion, inflammation, moisture, dressings, and ambient conditions [55,69].	Provides baseline context for interpreting wound and periwound measurements.	Not a therapeutic range. Wound-bed values can differ from surrounding skin and deeper tissue.
Approximately 36–38 °C: normothermic/supportive warming	Historical NNWT systems commonly used a dressing target near 38 °C; periwound warming has been measured during treatment [24,26].	Supports the rationale for perfusion and oxygen-delivery support. Re-epithelialization and fibroblast/keratinocyte repair are relevant biological endpoints, but a process-specific chronic-wound temperature threshold has not been established [75].	Human evidence is small and device-specific; actual wound-interface temperature is often incompletely mapped. The wIRA in-vitro evidence for keratinocyte/fibroblast repair [75] should not be interpreted as defining a validated 36–38 °C re-epithelialization window.
Approximately 39–42 °C: higher local warming/mild hyperthermia context	In intact skin, local heating in this range produces marked cutaneous vasodilation; heat-shock and immune responses are temperature- and time-dependent [5,57,60,64].	May increase perfusion and engage heat-shock and immunomodulatory pathways. In a microwave-based diabetic-mouse wound model, treatment was associated with M2-like macrophage polarization, neovascularization/angiogenesis, epithelial migration, and collagen remodeling [63].	The cited immunomodulatory and repair findings are model- and modality-specific; the microwave-treated diabetic-mouse study [63] does not establish a validated 39–42 °C chronic-wound threshold for angiogenesis, re-epithelialization, or matrix remodeling. Chronic-wound dose–response, safety, and optimal exposure duration remain unresolved.
Above approximately 42–43 °C or prolonged exposure	Thermal-stress and tissue-injury risk rises with temperature and time; CEM43 was developed in hyperthermia/cytotoxic contexts [42,43,73,74]. Repeated experimental skin heating can also increase MMP expression and collagen/extracellular-matrix damage [65].	Primarily relevant to safety limits and avoidance of excessive heating; matrix-remodeling effects may become adverse rather than reparative when thermal stress is excessive or repeated [65].	No validated universal injury threshold exists for ischemic, neuropathic, or heterogeneous chronic-wound tissue, and the experimental matrix findings should not be converted into a wound-specific numerical cutoff.

Abbreviations: CEM43, cumulative equivalent minutes at 43 °C; MMP, matrix metalloproteinase; NNWT, noncontact normothermic wound therapy; wIRA, water-filtered infrared A. Temperature ranges are overlapping operational categories used for synthesis in this review, not validated chronic-wound therapeutic or safety thresholds. Biological effects depend on exposure duration, measurement location, perfusion, moisture, and modality. The table explicitly identifies angiogenesis/neovascularization, immunomodulation, re-epithelialization, and matrix remodeling where supported, but it does not assign these processes to fixed therapeutic temperature thresholds when the available evidence is modality-specific, preclinical, or lacks wound-interface temperature measurements.

**Table 4:** Classification of temperature-related wound technologies by mechanism, intended role, control, and evidence context.

Modality/Platform	Energy Transfer/Primary Mechanism	Intended Role	Typical Temperature Context	Sensing/Control	Evidence Context and Critical Distinction
Noncontact normothermic or radiant wound therapy (NNWT)	Radiant heat from an external heater card through a noncontact wound cover	Maintain a warm, humid, near-normothermic wound environment	Historical systems commonly used a dressing target near 38 °C [24,26].	Fixed or thermostatic control; limited distributed interface sensing	Direct human chronic-wound studies exist; evidence is device-specific and should not be generalized to newer heater classes.
Water-filtered infrared A (wIRA)	Filtered infrared-A plus visible-light irradiation absorbed in tissue	Increase tissue temperature/perfusion with modality-specific optical effects	Defined by irradiation protocol rather than a single universal setpoint	External irradiation; temperature monitoring varies	Human venous-ulcer RCT exists [12], but wIRA has thermal and nonthermal/nonthermic effects, so healing cannot be attributed to heat alone.
Microwave exposure	Electromagnetic energy absorption in tissue	Experimental thermal/immune modulation	Device- and model-specific	External generator	Preclinical diabetic-wound evidence [63]; not direct clinical evidence for chronic-wound warming.
Flexible liquid-metal Joule heater	Resistive heating in deformable liquid-metal traces	Conformal programmable surface warming	Device-specific; chronic-wound therapeutic window not clinically established	Closed-loop control feasible	Engineering/preclinical evidence; encapsulation integrity and dose stability are central [8,87,88,89].
Graphene or metallic thin-film Joule heater	Resistive thin-film heating	Rapid conformal surface warming	Device-specific	Sensor and wireless integration feasible	Mostly bench/on-skin evidence; cracking or delamination can create local resistance changes and hot spots [9,90,91,92,93,94].
Hydrogel-integrated resistive heater	Conductive hydrogel or embedded resistive element	Close-contact warming, sometimes combined with drug delivery	Device-specific	Sensors can be embedded	Preclinical/engineering evidence; hydration state can change electrical and thermal output [92,95,96,97].
Transient or bioresorbable heater	Degradable resistive conductor	Time-limited heating or heat-triggered delivery	Device-specific and potentially time-varying during degradation	Often preset; limited feedback in current prototypes	Proof-of-concept evidence; degradation can alter electrical continuity and output [98,99,100,101].
Photothermal material or dressing	Optical absorption converted to heat, often combined with antimicrobial or drug mechanisms	Localized light-triggered treatment	Localized/transient; depends on irradiance, absorber, exposure time, and tissue optics	Feedback possible; requires external light delivery	Predominantly preclinical. PTT/PDT and drug-release systems are multimodal and are not equivalent to normothermic warming [52,53,102,103].
Temperature-monitoring device	Sensing only; no therapeutic heat delivery	Detect local temperature change or support risk monitoring	Passive measurement	Monitoring/alerts	Useful for assessment and prevention, but monitoring evidence is not evidence that therapeutic heating improves chronic-wound healing [104,105,106,107].
Wireless closed-loop thermotherapeutic dressing	Usually Joule heating integrated with temperature sensing, software, and communication	Measured, programmable interface dosing	Setpoint is device-specific	Closed-loop control, logging, and remote functions	Prototype/early translational architecture; control complexity introduces packaging, fault-handling, usability, and cybersecurity requirements [44,108,109].

Abbreviations: NNWT, noncontact normothermic wound therapy; PDT, photodynamic therapy; PTT, photothermal therapy; RCT, randomized controlled trial; wIRA, water-filtered infrared A.

**Table 5:** Device-specific regulatory and validation considerations for representative thermotherapeutic wound technologies.

Representative Device Example/Archetype	Established Regulatory and Standards Framework Relevant to the Device	Classification or Evidence Issue	Authors’ Proposed Wound-Interface Validation Criteria for Chronic-Wound Translation
Conventional powered heating pad or radiant warming device	US powered heating pads: 21 CFR 890.5740 [15]. IEC 60601-1 and, when applicable to the product type, IEC 60601-2-35 [16,134].	Intended use over a chronic-wound dressing may differ from conventional warming of intact skin; labeling and contraindications must match the use environment.	Measured interface temperature, peak and spatial variation, performance through intended dressings, moisture exposure, contact-pressure effects, and burn-risk controls.
Flexible resistive dressing with embedded temperature sensing	IEC 60601-1/-1-2; ISO 14971; IEC 60601-1-10 where a physiologic closed-loop controller is used [17,134,135,136]. EU MDR classification depends on intended purpose and energy hazard [130,131].	US pathway depends on intended use, technological characteristics, and availability of an appropriate predicate; a pathway should not be inferred solely from heater type.	Wet mechanical cycling, electrical isolation, sensor accuracy and drift, heater-sensor separation, control overshoot, fault handling, and spatial thermal mapping.
Externally activated photothermal hydrogel or scaffold	No universal regulatory class follows from the term ‘photothermal hydrogel’ or ‘photothermal scaffold’ alone. Classification depends on the material/device configuration, external light source, intended purpose, mode of action, and whether another regulated constituent contributes materially to the therapeutic action [130,131,132].	The regulatory analysis must consider both the externally delivered optical energy and the tissue-contacting material. Material biocompatibility/retention and multimodal PTT/PDT or drug effects can alter the evidence needed and complicate attribution of benefit to heat alone.	Calibrated irradiance and exposure, tissue-facing temperature map, optical attenuation through dressings/tissue, material retention/clearance, cytotoxicity, and separation of thermal from nonthermal effects.
Wireless closed-loop thermotherapeutic dressing	IEC 60601-1/-1-2 and, where applicable, IEC 60601-1-10; FDA cybersecurity guidance and IEC 81001-5-1 for connected functions; human-factors engineering [135,137,138,139]. Under the EU MDR, software and integrated closed-loop functions can introduce additional classification considerations depending on their intended purpose and role [130,131].	Software, communications, power, remote-control, and closed-loop functions expand the regulatory and safety analysis beyond heater output alone; the pathway cannot be inferred solely from the dressing or heater hardware.	End-to-end sensor/control verification, safe-state behavior, latency, alarm performance, authentication/update controls, usability under dressing changes, and long-wear moisture/pressure testing.
Heating plus drug-delivery combination dressing	Combination-product or medicinal-product/device boundaries depend on intended action, constituent roles, primary mode of action, and jurisdiction [132]. ISO 14971 and applicable electrical/software standards remain relevant to device functions.	The applicable pathway cannot be assigned from the presence of a heater or drug reservoir alone. Heat may alter release kinetics, tissue exposure, or constituent stability; evidence must address both components, their interaction, and the therapeutic role of each constituent.	Temperature-dependent release kinetics, dose uniformity, stability, local/systemic exposure, interaction with exudate and dressing changes, and combined-failure scenarios.

Abbreviations: CFR, Code of Federal Regulations; EU, European Union; FDA, U.S. Food and Drug Administration; IEC, International Electrotechnical Commission; ISO, International Organization for Standardization; MDR, Medical Device Regulation; PDT, photodynamic therapy; PTT, photothermal therapy. The regulatory provisions and standards summarized in the second column are established frameworks or requirements whose applicability depends on the specific product, intended purpose, jurisdiction, and device configuration. The final column presents wound-interface validation criteria proposed by the authors of this review to support chronic-wound translation, alongside the product-specific regulatory assessment required for each technology archetype.

**Table 6:** Quantitative outcome signals, thermal-dose limitations, and interpretation of direct clinical thermotherapy studies.

Study	Quantitative Outcome Signal	Thermal-Dose/Methodological Limitation	Interpretation
Robinson and Santilli, 1998 [18]	The abstract reports greater healing and a significant reduction in wound pain with Warm-Up therapy, but does not provide a numerical effect estimate.	Very small sample; arm sizes and achieved interface temperature not reported in abstract.	Hypothesis-generating venous-ulcer signal only.
Santilli et al., 1999 [19]	At the patient level, 14/17 patients improved during the 2-week inpatient trial; 8/17 healed completely after discharge; 1 recurrence occurred during 18-month follow-up.	Uncontrolled series; 31 wounds in 17 patients; interface temperature not reported.	Shows feasibility and a treatment signal but cannot establish comparative efficacy.
Price et al., 2000 [21]	Reported time advantage to 75% of original area: 6.4 days (*p* = 0.057); to 50%: 9.6 days (*p* = 0.039); to 25%: 7.2 days (*p* = 0.01).	Baseline imbalance despite randomization; dose details and attrition incompletely reported in abstract.	Suggestive pressure-ulcer signal with substantial uncertainty.
Whitney et al., 2001 [24]	Healing rate was faster with NNWT (Mann–Whitney U = 47, *p* = 0.01). Periwound temperature increased by 2.4 °C after 1 h (*p* = 0.001).	29/40 completed; measured periwound temperature does not provide full wound-bed spatial dose.	Provides clinical and mechanistic signal, but attrition and limited interface dosimetry constrain inference.
Kloth et al., 2002 [26]	Healing rate 0.52 versus 0.23 cm^2^/week (*p* < 0.02). Among wounds completing 12 weeks, closure was 11/14 (79%) versus 8/16 (50%), not statistically significant.	Wound-level analysis in 40 patients with 43 ulcers; protocol-completer closure denominator smaller than randomized wound count; interface dose mapping incomplete.	One of the stronger historical NNWT comparisons, but not practice-defining.
Alvarez et al., 2003 [27]	At 12 weeks, 70% of NNWT-treated wounds versus 40% of controls were healed in the interim *n* = 20 analysis; mean percent closure favored NNWT over time.	Interim, open-label, very small analysis; exact wound-interface temperature not reported.	Promising diabetic-foot signal requiring a larger adequately powered trial.
Karr, 2003 [28]	Mean healing time 59 days with NNWT versus 127 days with standard care; mean difference 68 days. The published analysis reported *p* = 0.33 for between-group healing-time difference.	Only 5 treatment patients/6 ulcers versus 11 controls/11 ulcers; the healing-time comparison is interpreted at the ulcer level because patient and wound counts differ in the treatment arm. Osteomyelitis treatment and surgery are major co-interventions; unequal groups and limited thermal dosimetry.	Exploratory osteomyelitis-associated wound evidence; the numerical mean difference should not be interpreted as definitive efficacy.
Thomas et al., 2005 [29]	Complete healing: 57% in the experimental group versus 44% in the control group (*p* = 0.46).	Small pilot; exact interface temperature and complete attrition/disposition details not reported in abstract.	Neutral/uncertain result; does not establish benefit.
Schumann et al., 2011 [12]	At 9 weeks, healing VAS median 85 versus 67.5 (median difference 15, 95% CI 3–30, *p* = 0.012); healing trend 21/25 (84%) versus 13/26 (50%), *p* = 0.023. The prespecified main variable did not reach significance.	wIRA + VIS is multimodal and includes nonthermal/nonthermic effects; wound-area reduction showed trends rather than definitive between-group significance.	Supports a venous-ulcer treatment signal for wIRA within compression/phlebological care, not for heat alone or other heater platforms.
Cherry and Wilson, 1999 [20]	Four of five patients healed completely during 12-week follow-up; granulation increased and pain decreased in all but one patient.	Uncontrolled *n* = 5 series; concurrent compression therapy prevents attribution to warming alone.	Feasibility and healing signal only; not comparative efficacy evidence.
Kloth et al., 2000 [22]	Mean wound-surface-area reduction was 60.73% with heated dressing plus standard care versus 19.24% with standard care alone; the heated-group reduction was statistically significant.	Small, nonrandomized controlled trial; 21 ulcers in 20 patients; wound-level analysis and short follow-up.	Supports a pressure-ulcer treatment signal, but design limitations preclude definitive efficacy conclusions.
Biland and Barras, 2001 [23]	The accessible primary citation is too brief for reliable quantitative extraction in this review.	Primary report detail is insufficient for independent extraction of group sizes, dosing, attrition, and outcome statistics.	Retained as core modality-specific treatment evidence, but quantitative interpretation is limited to what can be verified from the primary report.
McCulloch and Knight, 2002 [25]	Full-text journal version reports mean healing rate 0.019 ± 0.019 cm^2^/day with warming + offloading versus 0.008 ± 0.009 cm^2^/day with offloading alone (*p* = 0.049); 13/18 (72%) versus 5/18 (28%) healed within 2 months (χ^2^ = 9.03, *p* = 0.003).	Controlled allocation rather than clearly documented randomization; exact achieved wound temperature and detailed adverse-event reporting are not provided.	Suggestive diabetic-foot treatment signal when warming is added to offloading; not practice-defining.
Alvarez et al., 2006 [30]	Quantitative outcome data are not reproduced because the accessible PubMed record contains no abstract and the full primary report was not available for independent extraction.	Report is indexed as an RCT, but detailed methodological and thermal-dose information is unavailable in the accessible record.	Bibliographically verified treatment report; no quantitative claim is made beyond the accessible evidence.
Mercer et al., 2008 [31]	At the patient level, seven of 10 patients achieved complete or nearly complete ulcer healing and two additional patients had marked ulcer reduction during wIRA + VIS treatment, as reported in the primary article.	The study included 11 ulcers in 10 patients; the main variable was percent change in ulcer size over time, while some outcomes were summarized by patient. Only one patient could receive the VIS control condition, so the study functions largely as a prospective cohort and cannot establish comparative efficacy.	Supports feasibility and a treatment signal for wIRA + VIS in recalcitrant venous ulcers, not heat-alone efficacy.
Petrofsky et al., 2010 [32]	After 1 month, local heat alone reduced wound area by 30.1 ± 6.7% and wound volume by 22.3 ± 5.3%; the reductions were significant versus baseline. With heat + electrical stimulation, wound area and volume decreased by 68.4 ± 28.6% and 69.3 ± 27.1%, respectively (both *p* < 0.05 versus baseline); healing was significantly greater with heat + ES than with heat alone (*p* < 0.05).	Both groups received heat, so the study demonstrates a heat-only treatment arm but does not compare heat with no heat.	Shows measurable healing during local dry heat and additional benefit with ES; does not by itself establish heat efficacy versus standard care.

Abbreviations: CI, confidence interval; ES, electrical stimulation; NNWT, noncontact normothermic wound therapy; RCT, randomized controlled trial; VAS, visual analogue scale; VIS, visible light; wIRA, water-filtered infrared A. Note: Randomized design terminology in Table 6 and Supplementary Tables S2 and S3 reflects the description in the cited primary publication or, where stated, bibliographic indexing; it does not imply that the study met the eligibility or methodological criteria applied by Yue et al. [14].

**Table 7:** Potential indications, cautions, and knowledge gaps for thermotherapy in common wound scenarios.

Wound Context	Potential Role	Main Cautions	Evidence Position	Key Gap
Venous leg ulcers	May support perfusion and granulation	Heat excess, discomfort, compression interaction	Possible benefit in selected cases	Optimal dose with compression
Pressure ulcers/pressure injuries	May support local perfusion and repair	Pressure persistence, fragile skin, uneven heating	Limited evidence from small studies	Safe dosing under load
Diabetic foot ulcers (neuropathic)	Possible role in selected nonischemic wounds	Neuropathy, burn risk, occult ischemia, infection	Limited evidence; safety concerns significant	Safe thresholds and selection criteria
Ischemic/neuro-ischemic wounds	Uncertain role	Poor perfusion, thermal injury risk	Insufficient evidence for routine use	Perfusion thresholds for safe use
Infected wounds	Uncertain adjunct role	May worsen local risk if poorly controlled	Insufficient evidence	Interaction with infection and exudate
Mixed-etiology wounds	Possible role in carefully selected cases	Heterogeneous risk profile	Difficult to generalize	Stratified clinical trials
Post-debridement/periwound support	Possible short-term adjunct	Tissue viability and exudate concerns	Sparse evidence	Timing and duration
Home-use smart systems	May enable controlled personalized warming	User error, drift, adhesion failure, cybersecurity	Early promise, limited clinical validation	Long-wear safety and usability

**Table 8:** Single critical wound-interface failure mode and verification evidence for emerging thermotherapeutic platforms.

Platform	Current Evidence Context	Single Critical Wound-Interface Failure Mode	Evidence Needed to Address It	Relative Maturity
Liquid-metal heaters	Bench, on-skin, and small-animal engineering studies	Encapsulation or channel failure during wet cyclic deformation, causing leakage/electrical failure and loss of predictable heating.	Wound-fluid/humidity soak plus repeated bend/stretch/contact-pressure cycling; leak and electrical-isolation testing; resistance drift and spatial thermal mapping before and after cycling.	Preclinical
Graphene/metal thin-film heaters	Bench, on-skin, and early preclinical studies	Cracking or delamination that changes local resistance and creates hot spots or open circuits.	Wet cyclic flex/shear testing with adhesion assessment, electrical-resistance drift, infrared temperature mapping, and post-test defect inspection.	Preclinical
Hydrogel-integrated resistive heaters	Bench and small-animal studies	Swelling/drying-induced conductivity change causing output drift at the tissue interface.	Hydration, exudate, sterilization, and storage cycles with controlled-power and controlled-temperature tests; spatial temperature stability, sensor agreement, mechanical integrity, and leachable assessment.	Proof of concept
Transient/bioresorbable heaters	Proof-of-concept material/device studies	Degradation-driven change in electrical continuity and geometry, producing time-dependent dose drift.	Time-resolved electrical/thermal output during degradation in wound-relevant fluids; spatial temperature mapping; degradation chemistry, local cytotoxicity, and by-product exposure assessment.	Proof of concept
Photothermal materials	Bench and infected-wound animal studies	Nonuniform or poorly characterized optical energy deposition, causing local overheating or underdosing and confounding thermal with photodynamic/drug effects.	Calibrated irradiance, exposure time, and temperature maps through intended dressings/tissue models; optical attenuation, peak/spatial thermal spread, and separate reporting of ROS/drug mechanisms and material retention.	Proof of concept
Wireless smart thermotherapeutic dressings	Prototype/early feasibility studies	Packaging or power/control failure during long-wear moisture exposure, leading to incorrect sensor reading or unsafe heater command.	Multi-day wet mechanical testing with sensor drift, power stability, communication latency, safe-state/fault injection, alarm verification, and end-to-end thermal-control performance.	Early translational

Abbreviation: ROS, reactive oxygen species. Evidence categories refer to the thermotherapeutic function. The listed failure mode identifies the priority threat to wound-interface dose validity for each platform, while material, manufacturing, biocompatibility, electrical, and regulatory risks remain part of the broader validation pathway.

**Table 9:** Priority research questions and proposed approaches for advancing thermotherapeutic wound devices.

Priority Area	Key Question	Suggested Method	Core Measures	Translation Impact
Mechanisms in wound beds	Temperature–time effects on cells/ECM/immune signaling	Ex vivo tissue; pre/post biopsies	Perfusion/O_2_, cytokines, HSPs, MMP/ECM	Defines therapeutic window
Wound-specific dose models	Dose in moist, ischemic, heterogeneous wound beds	Bioheat and moisture models, in vivo validation	Predicted vs. measured temperature; CEM43 alternatives	Rational protocols and dose justification
Manufacturing and durability	Can devices survive real use?	Moisture/pressure cycling, fatigue tests	Thermal/electrical drift; failures	Industrialization and safer devices
High-risk limbs safety	Thresholds in neuropathy/ischemia	Stratified feasibility/safety studies	Burns/necrosis, perfusion change, PROs	Clinical guidelines and criteria
Combined modalities	Interactions with compression, NPWT, and antimicrobials	Factorial/add-on trials, bench under load	Healing rate, infection, under-device temperature	Integrated protocols; synergy versus harm
Standardized reporting	Make trials comparable	SPIRIT, CONSORT, TIDieR, and core outcomes [156,157,158,211]	Dose reporting, wound type, follow-up	Stronger evidence base
Standards for hybrids	What tests are missing?	Consensus and round-robin testing	Moisture/pressure tests; algorithm verification and validation	Clearer regulatory path
Remote monitoring	Safe home use and data workflows	Human factors and implementation pilots	Adherence, alerts, errors, security	Scales to home/community
Adaptive control	Can algorithms personalize safely?	Hardware-in-loop, monitored feasibility	Overshoot, stability, response to perfusion/moisture	Personalized dosing and regulatory confidence

Abbreviations: CEM43, cumulative equivalent minutes at 43 °C; CONSORT, Consolidated Standards of Reporting Trials; ECM, extracellular matrix; HSP, heat shock protein; MMP, matrix metalloproteinase; NPWT, negative-pressure wound therapy; O_2_, oxygen; PRO, patient-reported outcome; SPIRIT, Standard Protocol Items: Recommendations for Interventional Trials; TIDieR, Template for Intervention Description and Replication.

## Data Availability

No new data were created or analyzed in this study. Data sharing is not applicable to this article.

## Supplementary Materials

The following supporting information can be downloaded at: https://www.mdpi.com/article/10.3390/bioengineering13101126/s1, Table S1: Consolidated database-specific search expressions corresponding to the iterative search framework; Figure S1: Focused clinical-evidence selection flow for the direct-treatment synthesis; Table S2: Direct human studies of local warming for established chronic wounds: design, sample, thermal intervention, target/setpoint and achieved/measured temperature, follow-up, attrition, and adverse-event reporting; Table S3: Study-level qualitative risk-of-bias appraisal for direct clinical thermotherapy reports.
