# Supplementary material for: Thermotherapy for Chronic Wounds: A Wound-Interface Translation Framework for Materials, Devices, Clinical Evidence, and Validation Requirements
Source: Bioengineering (Basel). 2026 Sep 27;13(10):1126. doi: 10.3390/bioengineering13101126 (PMC13701685; doi:10.3390/bioengineering13101126)
Supplement: Supplementary file 1 [file bioengineering-13-01126-s001.zip › bioengineering-4573752-supplementary.pdf]

Supplementary Materials

Thermotherapy for Chronic Wounds: A Wound-Interface Translation Framework for Materials, Devices, Clinical Evidence, and Validation Requirements

Sameer Ahmad Hasan, Bassam Al-Naami, Muhammad Al-Ayyad, Abdel-Razzak Al-Hinnawi, and Olga Korostynska

Manuscript ID: bioengineering-4573752

Supplementary Table S1. Consolidated database-specific search expressions corresponding to the iterative search framework

Search period: December 2025–August 2026. Final literature cutoff: 31 August 2026. Literature searching was performed iteratively and topic by topic across the four databases as the review themes developed. The expressions below provide consolidated, database-specific formulations of the documented search concepts and support reproducible rerunning of the search framework. For this narrative review, selection accounting is reported at the focused candidate-report level, with the 25-report clinical verification flow documented in Supplementary Figure S1 and study-specific disposition in Table 1 of the revised manuscript.

Database / search period	Consolidated reproducible search expression
PubMed (NLM)<br>Search period: December 2025–August 2026<br>Final cutoff: 31 August 2026	(("chronic wound*"[Title/Abstract] OR "nonhealing wound*"[Title/Abstract] OR "non-healing wound*"[Title/Abstract] OR "diabetic foot ulcer*"[Title/Abstract] OR "diabetic ulcer*"[Title/Abstract] OR "venous leg ulcer*"[Title/Abstract] OR "venous ulcer*"[Title/Abstract] OR "venous stasis ulcer*"[Title/Abstract] OR "pressure ulcer*"[Title/Abstract] OR "pressure injur*"[Title/Abstract] OR "arterial ulcer*"[Title/Abstract] OR "lower extremity ulcer*"[Title/Abstract] OR "leg ulcer*"[Title/Abstract] OR "Diabetic Foot"[MeSH Terms] OR "Leg Ulcer"[MeSH Terms] OR "Pressure Ulcer"[MeSH Terms] OR "Varicose Ulcer"[MeSH Terms]) AND (thermotherap*[Title/Abstract] OR "heat therap*"[Title/Abstract] OR "local heat"[Title/Abstract] OR "local heating"[Title/Abstract] OR "local warming"[Title/Abstract] OR "wound warming"[Title/Abstract] OR "wound heating"[Title/Abstract] OR "warming therap*"[Title/Abstract] OR "heated dressing*"[Title/Abstract] OR "warming dressing*"[Title/Abstract] OR "radiant heat"[Title/Abstract] OR "radiant warming"[Title/Abstract] OR "normothermic wound therap*"[Title/Abstract] OR "noncontact normothermic wound therap*"[Title/Abstract] OR "non-contact normothermic wound therap*"[Title/Abstract] OR "water-filtered infrared A"[Title/Abstract] OR wIRA[Title/Abstract] OR "infrared A"[Title/Abstract] OR hypertherm*[Title/Abstract] OR photothermal*[Title/Abstract] OR "near infrared"[Title/Abstract] OR "near-infrared"[Title/Abstract] OR "thermal dose"[Title/Abstract] OR CEM43[Title/Abstract] OR thermoresponsive[Title/Abstract] OR "temperature-responsive"[Title/Abstract] OR "flexible heater*"[Title/Abstract] OR "wearable heater*"[Title/Abstract] OR microheater*[Title/Abstract] OR electrothermal[Title/Abstract] OR "Joule heating"[Title/Abstract] OR "smart dressing*"[Title/Abstract] OR "bioelectronic dressing*"[Title/Abstract] OR "wireless dressing*"[Title/Abstract] OR "closed-loop"[Title/Abstract] OR "feedback control"[Title/Abstract] OR "temperature sensor*"[Title/Abstract] OR "thermal sensor*"[Title/Abstract] OR "Infrared Rays"[MeSH Terms] OR "Hyperthermia, Induced"[MeSH Terms]))

Database / search period	Consolidated reproducible search expression
Embase.com (Elsevier)<br>Search period: December 2025–August 2026<br>Final cutoff: 31 August 2026	(('chronic wound*':ti,ab,kw OR 'nonhealing wound*':ti,ab,kw OR 'non-healing wound*':ti,ab,kw OR 'diabetic foot ulcer*':ti,ab,kw OR 'diabetic ulcer*':ti,ab,kw OR 'venous leg ulcer*':ti,ab,kw OR 'venous ulcer*':ti,ab,kw OR 'venous stasis ulcer*':ti,ab,kw OR 'pressure ulcer*':ti,ab,kw OR 'pressure injur*':ti,ab,kw OR 'arterial ulcer*':ti,ab,kw OR 'lower extremity ulcer*':ti,ab,kw OR 'leg ulcer*':ti,ab,kw) AND (thermotherap*:ti,ab,kw OR 'heat therap*':ti,ab,kw OR 'local heat':ti,ab,kw OR 'local heating':ti,ab,kw OR 'local warming':ti,ab,kw OR 'wound warming':ti,ab,kw OR 'wound heating':ti,ab,kw OR 'warming therap*':ti,ab,kw OR 'heated dressing*':ti,ab,kw OR 'warming dressing*':ti,ab,kw OR 'radiant heat':ti,ab,kw OR 'radiant warming':ti,ab,kw OR 'normothermic wound therap*':ti,ab,kw OR 'noncontact normothermic wound therap*':ti,ab,kw OR 'non-contact normothermic wound therap*':ti,ab,kw OR 'water-filtered infrared A':ti,ab,kw OR wIRA:ti,ab,kw OR 'infrared A':ti,ab,kw OR hypertherm*:ti,ab,kw OR photothermal*:ti,ab,kw OR 'near infrared':ti,ab,kw OR 'near-infrared':ti,ab,kw OR 'thermal dose':ti,ab,kw OR CEM43:ti,ab,kw OR thermoresponsive:ti,ab,kw OR 'temperature-responsive':ti,ab,kw OR 'flexible heater*':ti,ab,kw OR 'wearable heater*':ti,ab,kw OR microheater*:ti,ab,kw OR electrothermal:ti,ab,kw OR 'Joule heating':ti,ab,kw OR 'smart dressing*':ti,ab,kw OR 'bioelectronic dressing*':ti,ab,kw OR 'wireless dressing*':ti,ab,kw OR 'closed-loop':ti,ab,kw OR 'feedback control':ti,ab,kw OR 'temperature sensor*':ti,ab,kw OR 'thermal sensor*':ti,ab,kw))
Web of Science Core Collection<br>Search period: December 2025–August 2026<br>Final cutoff: 31 August 2026	TS=(("chronic wound*" OR "nonhealing wound*" OR "non-healing wound*" OR "diabetic foot ulcer*" OR "diabetic ulcer*" OR "venous leg ulcer*" OR "venous ulcer*" OR "venous stasis ulcer*" OR "pressure ulcer*" OR "pressure injur*" OR "arterial ulcer*" OR "lower extremity ulcer*" OR "leg ulcer*") AND (thermotherap* OR "heat therap*" OR "local heat" OR "local heating" OR "local warming" OR "wound warming" OR "wound heating" OR "warming therap*" OR "heated dressing*" OR "warming dressing*" OR "radiant heat" OR "radiant warming" OR "normothermic wound therap*" OR "noncontact normothermic wound therap*" OR "non-contact normothermic wound therap*" OR "water-filtered infrared A" OR wIRA OR "infrared A" OR hypertherm* OR photothermal* OR "near infrared" OR "near-infrared" OR "thermal dose" OR CEM43 OR thermoresponsive OR "temperature-responsive" OR "flexible heater*" OR "wearable heater*" OR microheater* OR electrothermal OR "Joule heating" OR "smart dressing*" OR "bioelectronic dressing*" OR "wireless dressing*" OR "closed-loop" OR "feedback control" OR "temperature sensor*" OR "thermal sensor*"))

Database / search period	Consolidated reproducible search expression
IEEE Xplore Command Search<br>Search period: December 2025–August 2026<br>Final cutoff: 31 August 2026	Block A1: clinical/local-warming concepts:<br>("chronic wound*" OR "diabetic foot ulcer*" OR "venous leg ulcer*" OR "pressure ulcer*" OR "pressure injur*" OR "arterial ulcer*" OR "leg ulcer*") AND (thermotherap* OR "heat therap*" OR "local heating" OR "local warming")<br><br>Block A2: clinical/local-warming concepts:<br>("chronic wound*" OR "diabetic foot ulcer*" OR "venous leg ulcer*" OR "pressure ulcer*" OR "pressure injur*" OR "arterial ulcer*" OR "leg ulcer*") AND ("wound warming" OR "wound heating" OR "radiant heat" OR wIRA)<br><br>Block A3: clinical/local-warming concepts:<br>("chronic wound*" OR "diabetic foot ulcer*" OR "venous leg ulcer*" OR "pressure ulcer*" OR "pressure injur*" OR "arterial ulcer*" OR "leg ulcer*") AND ("normothermic wound therap*" OR "water-filtered infrared A")<br><br>Block A4: clinical/local-warming concepts:<br>("chronic wound*" OR "diabetic foot ulcer*" OR "venous leg ulcer*" OR "pressure ulcer*" OR "pressure injur*" OR "arterial ulcer*" OR "leg ulcer*") AND (hypertherm* OR photothermal* OR "thermal dose" OR CEM43)<br><br>Block B1: device/control concepts:<br>("chronic wound*" OR "diabetic foot ulcer*" OR "venous leg ulcer*" OR "pressure ulcer*" OR "pressure injur*" OR "arterial ulcer*" OR "leg ulcer*") AND ("flexible heater*" OR "wearable heater*" OR microheater* OR electrothermal)<br><br>Block B2: device/control concepts:<br>("chronic wound*" OR "diabetic foot ulcer*" OR "venous leg ulcer*" OR "pressure ulcer*" OR "pressure injur*" OR "arterial ulcer*" OR "leg ulcer*") AND ("Joule heating" OR "smart dressing*" OR "bioelectronic dressing*")<br><br>Block B3: device/control concepts:<br>("chronic wound*" OR "diabetic foot ulcer*" OR "venous leg ulcer*" OR "pressure ulcer*" OR "pressure injur*" OR "arterial ulcer*" OR "leg ulcer*") AND ("wireless dressing*" OR "closed-loop" OR "feedback control" OR "temperature sensor*" OR thermoresponsive)<br><br>For reproducible rerunning of the documented concepts, all seven command-search blocks are executed separately and their results combined before deduplication. Each block remains within the current IEEE Xplore Command Search limit of 25 search terms per search clause. These are consolidated expressions, not a historical execution log.

Search framework. The database strategies combine chronic-wound terms with thermal-therapy, local-warming, infrared/wIRA, thermal-dose, heater, smart-dressing, sensing, and feedback-control concepts. Searches were supplemented by backward citation tracking. Regulatory standards and guidance were identified separately from official regulatory and standards sources because these documents are not comprehensively indexed in bibliographic databases. The focused clinical-evidence verification is reported at report level and linked directly to the study-specific disposition in the revised manuscript.

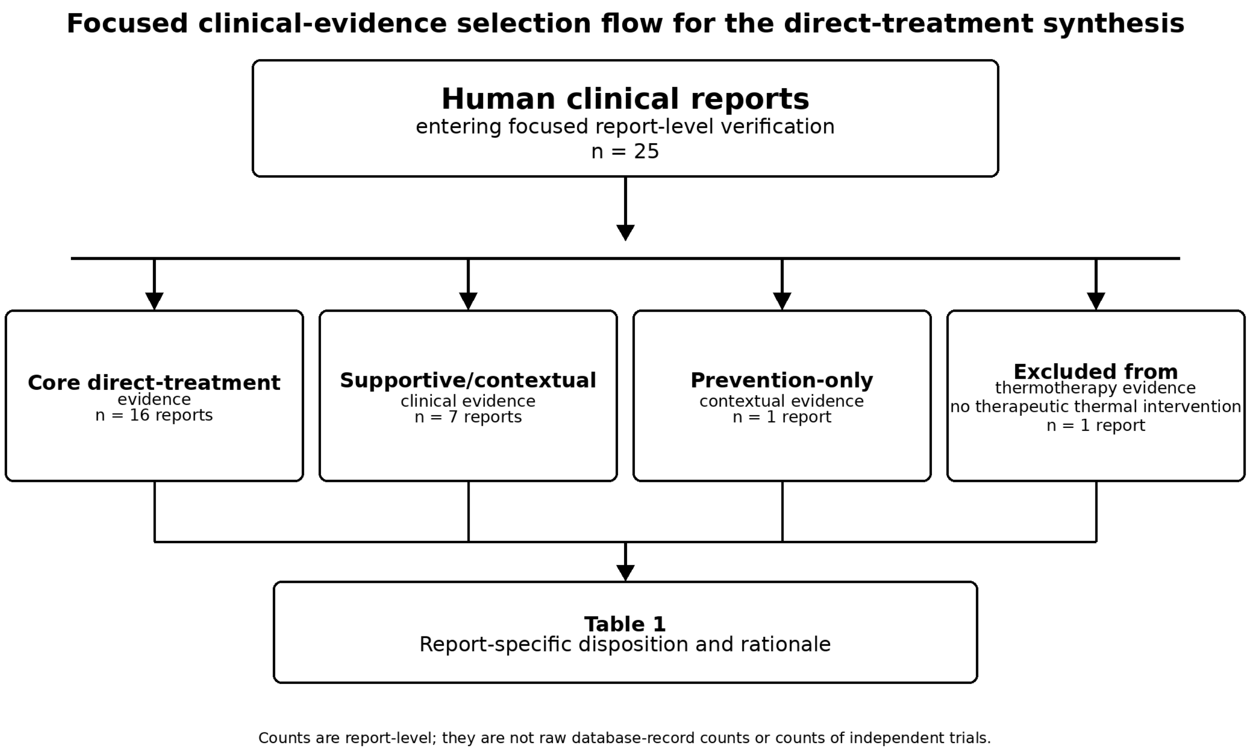

Supplementary Figure S1. Focused clinical-evidence selection flow for the direct-treatment synthesis.

Twenty-five unique human reports entered focused report-level verification. Sixteen met the criteria for the core direct-treatment synthesis; seven were retained as supportive/contextual clinical evidence; one prevention-only report was retained as contextual evidence; and one report was classified outside thermotherapy evidence because it did not evaluate a therapeutic thermal intervention. These four mutually exclusive dispositions account for all 25 candidate reports. The counts represent the focused report-level clinical evidence set. Study-specific disposition and rationale are provided in Table 1 of the revised manuscript. Supplementary Figure S1 presents the corresponding focused narrative-review evidence-selection flow.

Supplementary Table S2. Direct human studies of local warming for established chronic wounds: design, sample, thermal intervention, target/setpoint and achieved/measured temperature, follow-up, attrition, and adverse-event reporting.

Study	Participants, treatment/control group sizes, unit of analysis, and baseline wound characteristics	Design / comparator	Thermal intervention, target/setpoint, and achieved/measured temperature	Follow-up / attrition / adverse events
Robinson and Santilli, 1998 [18]	13 patients with chronic venous stasis ulcers; patient-level trial; arm sizes not reported in the abstract. Baseline ulcer size and chronicity are not reported in the indexed abstract.	Preliminary prospective study; patients were randomly assigned to conventional therapy or Warm-Up radiant-heat therapy.	Radiant-heat bandage; normothermic intent. Exact achieved wound-interface temperature and spatial mapping not reported in the abstract.	Follow-up duration is not reported in the indexed abstract; detailed attrition is not reported; no adverse events were reported.
Santilli et al., 1999 [19]	17 patients with 31 chronic venous stasis ulcers; mean ulcer duration 4.44 years; patients had failed aggressive inpatient and outpatient conventional therapy; multiple wounds were present in some patients. This is summarized as a patient cohort with multiple ulcers per patient; the 31 ulcers are not treated as 31 independent patients.	Prospective uncontrolled treatment series after failure of conventional therapy.	Noncontact radiant-heat bandage for 5 h/day: three 1-h heating periods separated by two 1-h nonheating periods for 2 weeks. Achieved interface temperature not reported in abstract.	2-week inpatient treatment with follow-up after discharge; 1 recurrence during 18 months; no adverse effects reported.
Price et al., 2000 [21]	58 participants were randomized; 50 were included in the analysis (25 radiant-heat, 25 comparator). Stage III–IV noninfected pressure ulcers; patient-level randomized trial as described in the primary report. Despite randomization, the radiant-heat group was more infirm at baseline; detailed wound-size comparability is not reported in the indexed abstract.	Prospective single-center randomized comparison with standard treatment, as described in the primary report.	Radiant heat dressing; exact target/achieved wound-interface temperature and spatial distribution not reported in abstract.	6-week follow-up; 50/58 randomized participants were included in the analysis. The accessible evidence summary does not specify a reason for each post-randomization exclusion. Adverse-event information is not reported in the indexed abstract.
Whitney et al., 2001 [24]	40 subjects enrolled with Stage III–IV pressure ulcers; 29 completed (15 NNWT, 14 standard care); participant-level randomized trial as described in the primary report. The publication reports that the groups were statistically similar with regard to baseline and wound characteristics; exact baseline wound-size values are not reproduced in the indexed abstract.	Prospective randomized 8-week trial, as described in the primary report; moist/absorptive standard dressings as comparator.	NNWT dressing set to 38 °C for 1 h, three times daily. Periwound temperature was measured and increased by a mean of 2.4 °C after 1 h of warming ($p=0.001$), reaching levels described by the authors as approximating normothermia. The indexed abstract does not provide an absolute achieved wound-bed temperature or spatial wound-interface map.	8 weeks; 11/40 did not complete; adverse events not reported in abstract.

Study	Participants, treatment/control group sizes, unit of analysis, and baseline wound characteristics	Design / comparator	Thermal intervention, target/setpoint, and achieved/measured temperature	Follow-up / attrition / adverse events
Kloth et al., 2002 [26]	40 inpatients with 43 chronic full-thickness Stage III–IV pressure ulcers; unit of allocation/analysis included wounds; 21 NNWT and 22 control wounds. Baseline wound-size values are not reported in the indexed abstract.	Prospective randomized controlled trial versus standard moisture-retentive dressings, as described in the primary report.	Noncontact dressing; heating element activated for three 1-h periods/day for up to 12 weeks or closure. Continuous interface mapping not reported.	Up to 12 weeks; closure analysis among protocol completers used 14 NNWT and 16 control wounds; complete disposition of the remaining randomized wounds is not reported ; adverse events not reported in abstract.
Alvarez et al., 2003 [27]	20 patients completed the interim analysis; 10 NNWT and 10 control. The accessible abstract reports the interim cohort at the participant level but does not state a separate wound count. Neuropathic diabetic foot ulcers; baseline demographics and ulcer size were not significantly different. Mean baseline ulcer area was 346 mm^2 in the NNWT group and 216 mm^2 in controls; forefoot location was 70% versus 80%, respectively.	Prospective randomized, parallel-group, open-label controlled interim analysis, as described in the primary report; both groups received debridement and offloading.	NNWT 1 h three times daily until healing or 12 weeks. The accessible primary abstract reports the treatment schedule but not a directly measured achieved wound-interface temperature.	12 weeks; interim completer analysis n=20 (10/arm); complete enrollment and attrition disposition before the interim cohort are not established from the accessible report; no NNWT-associated adverse events reported in this subset.
Karr, 2003 [28]	Control: 11 patients/11 ulcers; NNWT: 5 patients/6 ulcers; wounds overlying managed lower-extremity osteomyelitis. Because one treatment participant contributed more than one ulcer, patient and wound counts are not interchangeable; wound-level outcomes are identified as such. Mean pre-treatment ulcer duration was 267 days in controls and 391 days in the NNWT group.	Single-center prospective pilot; numbered-envelope randomization reported; both groups could receive debridement, osteomyelitis surgery/antibiotics and offloading as clinically indicated.	Warm-Up system as wound dressing in treatment arm; published report does not provide continuous wound-interface temperature mapping.	Weekly wound-care evaluations; followed to complete healing. No formal attrition rate was stated. No quantitative adverse-event information specific to the heating intervention was identified in the accessible report.
Thomas et al., 2005 [29]	41 participants with Stage III–IV truncal pressure ulcers >1 cm^2 were randomized; the indexed abstract describes each subject as having a qualifying pressure ulcer and reports healing by subject, but it does not provide a separate wound count. Of the reported ulcers, 22 were Stage III and 19 Stage IV. The exact initial treatment/control arm sizes are unavailable from the accessible primary record.	Controlled randomized comparative pilot, as described in the primary report; radiant-heat dressing versus hydrocolloid dressing with or without alginate filler.	Radiant-heat dressing; the indexed abstract does not report a directly measured achieved wound-interface temperature or cumulative temperature–time exposure.	Followed until healing or 12 weeks. Exact initial arm sizes and detailed attrition/adverse-event disposition are unavailable from the accessible primary record.

Study	Participants, treatment/control group sizes, unit of analysis, and baseline wound characteristics	Design / comparator	Thermal intervention, target/setpoint, and achieved/measured temperature	Follow-up / attrition / adverse events
Schumann et al., 2011 [12]	51 patients with nonhealing chronic venous stasis ulcers of the lower legs; wIRA+VIS n=25, VIS control n=26. The indexed abstract does not provide a complete numeric baseline wound-characteristic table, so unreported baseline values are unavailable in the accessible record.	Investigator-initiated randomized controlled blinded study, as described in the primary report; both groups received phlebological/compression therapy, cleansing, and dressings.	30 min irradiation, five times/week for 9 weeks; wIRA+visible light versus visible light alone. The intervention has thermal and nonthermal/nonthermic effects and is defined by an irradiation protocol rather than a single wound-interface temperature setpoint; an achieved wound-bed temperature is not reported in the indexed abstract.	9-week irradiation plus 4 weeks without irradiation; group sizes reported. Detailed attrition data require the full report and are unavailable in the indexed abstract. Adverse-event information is also not reported in the indexed abstract.
Cherry and Wilson, 1999 [20]	5 ambulatory patients with venous ulcers; mean ulcer duration 8 months (range 3–13 months); patient-level case series.	Uncontrolled treatment series; warming used with zip-up compression stockings.	Warming dressing controlled to a 38 °C treatment target for 1 h three times daily for 2 weeks. The indexed abstract does not report an independently measured achieved wound-bed temperature.	Healing monitored for 12 weeks; 4/5 ulcers healed. Attrition and adverse events were not reported in the indexed abstract.
Kloth et al., 2000 [22]	20 inpatients with 21 Stage III–IV pressure ulcers; 15 heated ulcers and 6 ulcers in a separate control group. The healing analysis is wound-level (21 ulcers in 20 patients). The indexed abstract does not report baseline wound-size values for the two groups.	Controlled before–after clinical trial; standard care plus heated dressing versus standard care alone.	Semiocclusive heated dressing set to 38 °C; two 60-min heating periods daily within a 4.5-h application, Monday–Friday for 4 weeks. Mean skin temperature increased by 0.97 °C within the heated-dressing region and by 1.08 °C adjacent to it from baseline ($p<0.05$). An absolute achieved wound-bed temperature and spatial wound-interface map were not reported in the indexed abstract.	4 weeks; the indexed abstract reports no adverse effects from the radiant heat dressing.
Biland and Barras, 2001 [23]	Patients with chronic venous ulcers; detailed participant, baseline wound-characteristic, and unit-of-analysis data are unavailable from the accessible primary citation.	Primary report is a brief publication; later wIRA reviews describe a controlled clinical comparison, but detailed primary-study appraisal remains limited.	wIRA-based treatment; a complete primary-report temperature–time record and achieved wound-interface temperature are unavailable from the accessible primary citation.	Follow-up, attrition, and adverse-event details are unavailable from the accessible primary citation.

Study	Participants, treatment/control group sizes, unit of analysis, and baseline wound characteristics	Design / comparator	Thermal intervention, target/setpoint, and achieved/measured temperature	Follow-up / attrition / adverse events
McCulloch and Knight, 2002 [25]	40 patients with neuropathic diabetic foot ulcers were allocated 20 per group; 36 were analyzed/completed (18 warming + offloading, 18 offloading-only). Baseline ulcer surface area was 2.02 ± 1.53 cm² in the warming group and 2.58 ± 2.89 cm² in controls; the difference was not statistically significant.	Controlled clinical study; offloading plus noncontact radiant warming versus offloading alone.	Noncontact warming system with a reported device target of 38 ± 1 °C; treatment for 3 h/day, 5 days/week, with the heater on for 1 h, off for 1 h, and on for a final 1 h; offloading was used in both groups. A directly measured achieved wound-bed temperature or spatial interface map was not reported in the accessible article.	Up to 8 weeks or until healing. Four of 40 allocated patients were removed for non-study-related issues (2 per group), leaving 18 participants per group for analysis. Detailed adverse-event reporting was not provided in the accessible article.
Alvarez et al., 2006 [30]	Diabetic neuropathic foot ulcers; PubMed confirms the report and indexes it as a randomized controlled trial, but no abstract is available for reliable extraction of participant-level or baseline wound-characteristic details; these are unavailable in the accessible record.	Randomized controlled trial according to PubMed indexing; detailed comparator and allocation data are not reproduced without an accessible primary report.	NNWT; detailed target/setpoint, achieved temperature, and temperature–time information are not available in the accessible PubMed record.	Follow-up, attrition, and adverse-event details are not available in the accessible PubMed record.
Mercer et al., 2008 [31]	10 patients (5 male, 5 female) with 11 recalcitrant chronic venous stasis ulcers of the lower legs; median age 62 years. Patient- and ulcer-level reporting coexist: healing is summarized by patient, whereas the primary outcome is percent change in ulcer size over time. Detailed baseline ulcer-size values are not summarized in the indexed abstract.	Prospective study originally planned as randomized, controlled and blinded; de facto, except for one patient, only one cohort was possible.	wIRA+VIS or VIS; 30-min irradiation two to five times/week at 25 cm; approximately 140 mW/cm² wIRA and 45 mW/cm² VIS. Infrared thermography was used during treatment. Typical skin temperatures during irradiation were 37–39 °C, while the ulcer base could remain below 37 °C; skin-surface temperature was not allowed to exceed 41 °C. These measurements show substantial spatial heterogeneity rather than a single wound-interface setpoint.	Up to 2 months, typically until closure or near-closure; comparative inference is limited by the de facto cohort design. Adverse-event reporting is not separately described in the accessible report.
Petrofsky et al., 2010 [32]	20 patients with single nonhealing diabetic foot ulcers; local heat n=10, local heat + electrical stimulation n=10. Patient and study-ulcer counts therefore coincide. All wounds were Wagner Grade 2; mean ulcer duration was 38.9 ± 23.7 months. Baseline wound area and volume did not differ significantly between groups.	Two active-treatment groups; heat-only versus heat + electrical stimulation. No no-heat control.	Local dry heat delivered by an infrared lamp three times/week for 4 weeks. A thermocouple measured skin temperature outside the wound and a controller adjusted lamp intensity to maintain 37 °C; the article describes warming the local skin from approximately 31–32 °C to 37 °C. Wound-bed temperature was not separately reported. Electrical stimulation was added in the second group.	4 weeks; indexed abstract reports wound-area outcomes and blood-flow measurements; adverse-event reporting is not described in the abstract.

Abbreviations: NNWT, noncontact normothermic wound therapy; VIS, visible light; wIRA, water-filtered infrared A. Study characteristics and outcomes are reported only where directly supported by the accessible primary publication or a directly traceable authoritative evidence extraction; unavailable items are identified explicitly.

Supplementary Table S3. Study-level qualitative risk-of-bias appraisal for direct clinical thermotherapy reports.

Study	Assignment / selection	Attrition and unit of analysis	Blinding / co-interventions	Thermal-dose measurement	Overall qualitative concern
Robinson and Santilli, 1998 [18]	Random assignment reported; details limited in abstract.	n=13; arm sizes and attrition details not reported in abstract.	Blinding not reported; conventional care comparator.	Achieved interface dose not reported.	High
Santilli et al., 1999 [19]	Uncontrolled selected inpatient series after prior treatment failure.	17 patients/31 wounds; multiple wounds per patient, creating potential within-patient clustering.	No control; concomitant wound care not separable from warming effect.	Achieved interface dose not reported.	High
Price et al., 2000 [21]	Randomization reported, but treatment group was more infirm at baseline.	58 randomized; 50 analyzed (25/arm); complete reasons for post-randomization exclusions are not reliably established from the accessible record.	Blinding not reported; standard care comparator.	Dose and spatial temperature incompletely reported.	High
Whitney et al., 2001 [24]	Randomization reported in the primary publication; baseline/wound characteristics reported as statistically similar.	40 enrolled; 29 completed (15 NNWT, 14 control); 11 did not complete; completion is reported at the participant level.	Blinding not reported; standard care comparator.	Periwound temperature increased by a mean 2.4 °C after 1 h, but absolute wound-bed temperature and spatial dose were not reported.	High
Kloth et al., 2002 [26]	Randomized controlled wound-level trial as described in the primary publication.	40 patients/43 wounds randomized (21 NNWT, 22 control); reported protocol-completer closure analysis used 14 versus 16 wounds; complete disposition of the remaining wounds is not reconstructed.	Blinding not reported; standard dressings in control.	Treatment schedule reported; continuous/spatial interface dose incomplete.	Some concerns to high
Alvarez et al., 2003 [27]	Computer-generated randomization reported; open-label interim analysis.	Interim completer cohort of 20 patients (10/arm); a separate wound count is not stated in the accessible report, and complete enrollment and attrition disposition are not established.	Both arms received debridement/offloading; open-label.	Exact achieved interface temperature not reported.	High
Karr, 2003 [28]	Numbered-envelope randomization reported; treatment arm stopped early after 5 patients.	11 control patients/11 ulcers vs 5 treatment patients/6 ulcers; unequal groups. One treatment participant contributed more than one ulcer, so patient and wound counts are not interchangeable and ulcer-level outcomes may be clustered within participant.	Osteomyelitis surgery, antibiotics, debridement and offloading could influence healing.	Continuous interface dose not reported.	High

Study	Assignment / selection	Attrition and unit of analysis	Blinding / co-interventions	Thermal-dose measurement	Overall qualitative concern
Thomas et al., 2005 [29]	Randomized comparison reported.	41 subjects; the indexed abstract describes each subject as having a qualifying pressure ulcer and reports healing by subject, but does not provide a separate wound count; complete disposition by arm is not reported.	Blinding not reported; hydrocolloid ± alginate control.	Exact achieved interface temperature not reported.	High
Schumann et al., 2011 [12]	Randomized controlled blinded study as described in the primary publication; 25 vs 26.	Group sizes reported; full attrition details not inferred from abstract.	Both groups received standard venous-ulcer care; active VIS comparator.	wIRA exposure is not defined by a single temperature and includes nonthermal effects.	Some concerns
Cherry and Wilson, 1999 [20]	Uncontrolled selected case series.	5 patients; patient-level follow-up; no comparator.	Compression therapy used concurrently; no blinding.	38 °C target and schedule reported; full spatial/interface dose not reported.	High
Kloth et al., 2000 [22]	Controlled before–after study; separate control group, not randomized.	20 patients/21 ulcers; 15 treated and 6 control ulcers; wound-level analysis.	Blinding not reported; standard wound care continued.	38 °C device setting; measured skin-temperature increases were +0.97 °C within and +1.08 °C adjacent to the heated dressing, but absolute wound-bed/spatial dose was not reported.	High
Biland and Barras, 2001 [23]	Detailed allocation method not reliably extractable from accessible primary citation.	Participant disposition and unit-of-analysis details not reliably extractable.	Co-interventions/blinding not independently verifiable from accessible primary citation.	Complete thermal-dose record not available from the accessible primary citation.	High / insufficient reporting
McCulloch and Knight, 2002 [25]	Initial group assignment was determined by coin toss, followed by alternating allocation for subsequent participants; therefore the allocation sequence was not fully randomized.	40 allocated; 36 completed/analyzed (18/arm); four participants were removed for non-study-related reasons, two per group.	Both groups offloaded; blinding not reported.	Device target 38 ± 1 °C and intermittent 3-h schedule reported; achieved wound-bed temperature and spatial interface mapping were not reported.	High
Alvarez et al., 2006 [30]	PubMed indexes the report as randomized controlled; allocation details not available in the accessible record.	Participant and attrition details not available in the accessible PubMed record.	Blinding/co-interventions not reliably extractable from the accessible record.	Detailed interface dose not available in the accessible PubMed record.	High / insufficient reporting

Study	Assignment / selection	Attrition and unit of analysis	Blinding / co-interventions	Thermal-dose measurement	Overall qualitative concern
Mercer et al., 2008 [31]	Randomized controlled design was planned, but de facto only one patient could receive the VIS comparison.	10 patients/11 ulcers; mixed patient- and ulcer-level reporting, with potential within-patient clustering because one participant contributed more than one ulcer; mostly single-cohort analysis.	wIRA+VIS is multimodal; standard wound care continued; comparative blinding could not be implemented as planned for most participants.	Thermography documented typical skin temperatures of 37–39 °C during irradiation, with ulcer-base temperatures sometimes below 37 °C; spatial heterogeneity remained substantial.	High
Petrofsky et al., 2010 [32]	Two active-treatment groups; random allocation not stated in the indexed abstract.	20 patients with single study ulcers, 10 per group; patient and wound counts coincide; 4-week treatment period.	No no-heat comparator; one group also received electrical stimulation.	Local skin outside the wound was feedback-controlled to 37 °C; wound-bed temperature and complete spatial interface dose were not reported.	High

Abbreviations: NNWT, noncontact normothermic wound therapy; VIS, visible light; wIRA, water-filtered infrared A.

This qualitative, domain-based appraisal summarizes study-level risk-of-bias concerns using methodological information reported in or reliably extractable from the available primary publications. The domains capture the principal threats to causal inference in this literature: assignment/selection, attrition and unit of analysis, blinding/co-interventions, and thermal-dose measurement. The overall qualitative categories provide a descriptive synthesis rather than a numerical score: "Some concerns" indicates important limitations without a clearly dominant major threat to causal inference; "High" indicates one or more major limitations that substantially restrict causal inference; "High / insufficient reporting" indicates major limitations together with substantial gaps in the accessible methodological record; and "Some concerns to high" denotes an intermediate descriptive judgment. These categories support interpretation of the reported observations and are distinct from a formal RoB 2 assessment.
